# Spatially programmed regioisomeric conjugated microporous polymers modulating zinc sites for selective CO_2_ photoreduction to CH_4_†

**DOI:** 10.1039/d5sc02835c

**Published:** 2025-06-30

**Authors:** Xingwang Lan, Juan Wang, Lu Chen, Haobo Xu, Tianjun Zhang, Yong Chen

**Affiliations:** a College of Chemistry and Materials Science, Key Laboratory of Chemical Biology of Hebei Province, Hebei Research Center of the Basic Discipline of Synthetic Chemistry, Institute of Life Science and Green Development, Hebei University Baoding Hebei 071002 P.R. China lanxingwang@hbu.edu.cn; b Key Laboratory of Photochemical Conversion and Optoelectronic Materials & CAS-HKU Joint Laboratory on New Materials, Technical Institute of Physics and Chemistry, Chinese Academy of Sciences Beijing 100190 P. R. China chenyong@mail.ipc.ac.cn; c University of Chinese Academy of Sciences Beijing 100049 P. R. China

## Abstract

Conjugated microporous polymers show great potential for photocatalytic CO_2_ reduction into value-added products. However, their catalytic activity and selectivity remain significantly limited due to poor charge separation efficiency and the lack of suitable active sites. Herein, we propose a topology-driven dipole programming strategy that synergistically decouples atomic-level electronic configuration control from spatially resolved active site engineering. Crucially, the regioisomer-dependent π-topology governs light-harvesting ability, dipole polarization hierarchy, and directional charge transport networks. As a result, the designed Zn-TPA-BPy-1, featuring dipole polarization fields and Zn–N_2_O_2_ sites, exhibits exceptional photocatalytic CO_2_ conversion activity, with a CH_4_ evolution rate of 753.18 μmol g^−1^ h^−1^ and a high selectivity of 89.7%. Experimental and theoretical results reveal that asymmetric dipole arrays lower the energy barrier for *COOH and *CO intermediates while stabilizing *CHO intermediates through dynamic charge compensation, which contribute to the high activity and selectivity. This finding offers new insights into designing polymer-photocatalysts by subtle structural modulation for CO_2_ conversion.

## Abstract 2

A topological spatial engineering strategy was proposed to precisely regulate dipole polarization and active sites in conjugated microporous polymers, achieving high photocatalytic CO_2_-to-CH_4_ conversion activity and selectivity.

## Graphical abstract

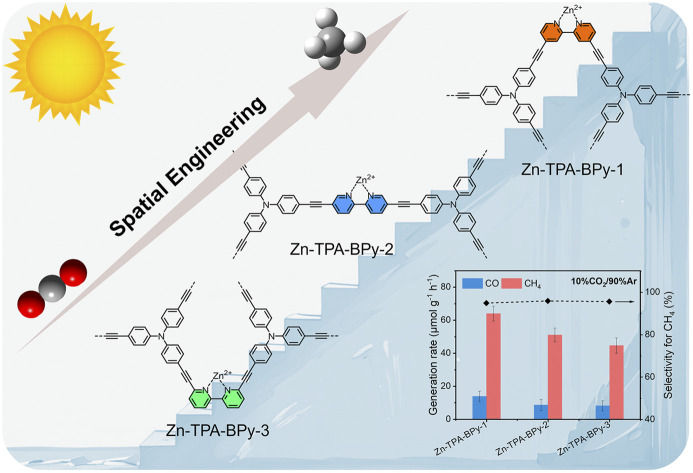

## Introduction

The photocatalytic conversion of carbon dioxide (CO_2_) into value-added fuels is a promising technology for mitigating emerging environmental crises and achieving carbon neutrality.^1^ Among the diverse C_1_ products (*e.g.*, CO, HCOOH, CH_3_OH, CH_4_, *etc.*) derived from CO_2_ reduction, methane (CH_4_) is attracting significant research interest due to its high enthalpy of combustion and could replace natural gas.^2^ Nevertheless, CH_4_ production usually entails multiple proton-coupled electron transfer processes with eight electrons and reactive intermediates,^3^ which is kinetically sluggish relative to the competing two-electron CO_2_-to-CO process, leading to the low activity and selectivity of CH_4_ formation.^4^ Therefore, to realize efficient and selective CO_2_ reduction towards CH_4_ products, designing photocatalysts that can manipulate the binding strengths of C_1_ intermediates and enable preferential CH_4_ formation^5^ is highly desirable and imperative but remains challenging.

**Fig. 1:**
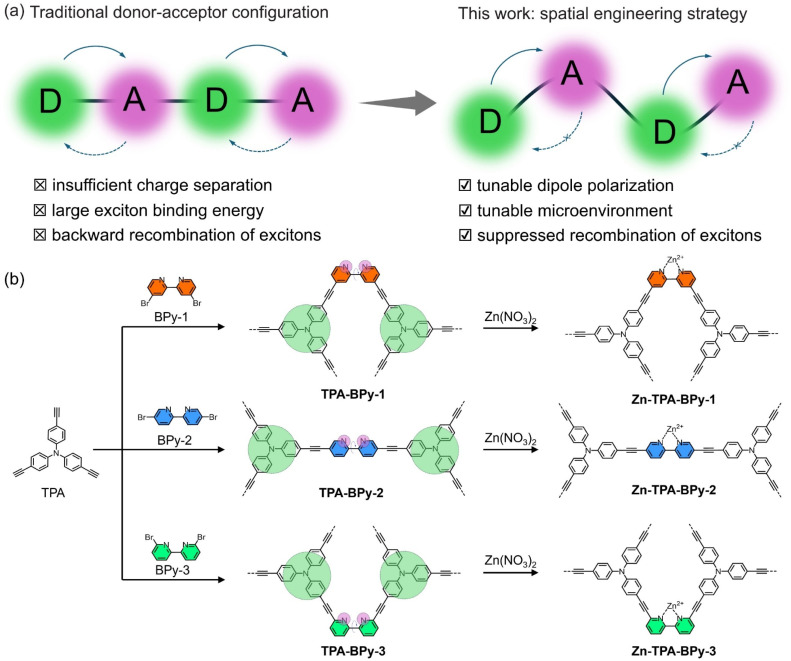
(a) Scheme of the traditional donor–acceptor configuration and the design strategy for spatial engineering. (b) Synthetic routes of anchoring Zn species of TPA-BPy-1, TPA-BPy-2, and TPA-BPy-3.

Generally, photocatalytic CO_2_ reduction involves three vital processes: light harvesting, charge carrier separation, and CO_2_ adsorption and activation.^6^ Accordingly, ideal photocatalysts should supply sufficient photogenerated charges and possess efficient carrier transfer channels to facilitate the direct transfer of electrons toward the active sites. Recently, porous conjugated polymers, including conjugated microporous polymers (CMPs)^7–10^ and covalent organic frameworks (COFs),^11–16^ have shown great potential as catalysts or supports for CO_2_ photoreduction owing to their unique advantages originating from high porosity, outstanding stability, and adjustable π-conjugated structure. Particularly, the donor–acceptor (D–A) configuration materials have attracted more attention because of the prominent photon-capture ability and the electronic pull–push effect, accelerating the splitting of photogenerated excitons.^17^ However, for the numerous reported D–A systems, large exciton binding energy and backward recombination of excitons still impact sufficient charge transfer to the surface,^18–20^ which negatively limits their CO_2_ photoreduction capability (Fig. 1a). To overcome these challenges and engineer D–A systems with enhanced photoactivity, dipole field engineering has been employed to modulate charge transport dynamics through strategic manipulation of donor and acceptor moieties.^21,22^ Generally, the high dipole moments formed by the distribution of electron clouds can enhance internal polarization,^23^ resulting in narrow energy gaps with red-shifted absorption and emission properties, promoting the directed charge separation during redox reactions.^24^ Although molecular-level dipole modulation through building unit modification has achieved preliminary success, strategic spatial engineering in topological networks for directional charge transfer is rare. Particularly noteworthy is the insufficient exploration of these dipole engineering principles in CO_2_ photoreduction systems. Furthermore, most organic polymeric materials suffer from the absence of catalytic active sites, leading to unsatisfactory photocatalytic performance.^25^ To achieve high efficiency and tailored catalytic performance, various catalytic metal centers and linker molecules have been assembled into the polymer materials.^26–28^ However, the key bottleneck to effectively drive CO_2_ photoreduction lies in precisely arranging the location of catalytic atoms and finely regulating the electronic state of the metal site environment.

With these considerations in mind, we propose a topological spatial engineering strategy to achieve atomic-level modulation in electronic structure tailoring and active site localization. Through regiochemical control over bipyridine substituents in modular tris(4-ethynylphenyl)amine precursors, a family of regioisomeric alkynyl-linked CMPs, namely, TPA-BPy-*n* (*n* = 1, 2, 3) were synthesized (Fig. 1b). The strategic positioning of donor-π-acceptor (D–π–A) motifs with pronounced intramolecular charge transfer (ICT) characteristics enabled systematic control over framework dipole moments, while spatial orientation precisely determines whether metal coordination occurs inside or outside the channels. Based on these, the atomically dispersed Zn sites were incorporated into bipyridine units of TPA-BPy-*n* for photocatalytic CO_2_ reduction. Photocatalytic evaluations revealed that single Zn sites located outside the channels of TPA-BPy-1, possessing the longest dipole moment, exhibited higher kinetic activity, CH_4_ and CO production-rates at 753.2 and 233.9 μmol g^−1^ h^−1^, respectively, and the CH_4_-product selectivity at 89.7%. Combined with the experimental and theoretical results, we demonstrated that the effects of intramolecular dipole polarization and microenvironment can effectively modulate catalytic performances. This work highlights that the topology-driven dipole programming strategy can modulate the electronic structure of active sites to promote photocatalytic CO_2_ reduction.

## Results and discussion

### Synthesis and characterization

**Fig. 2:**
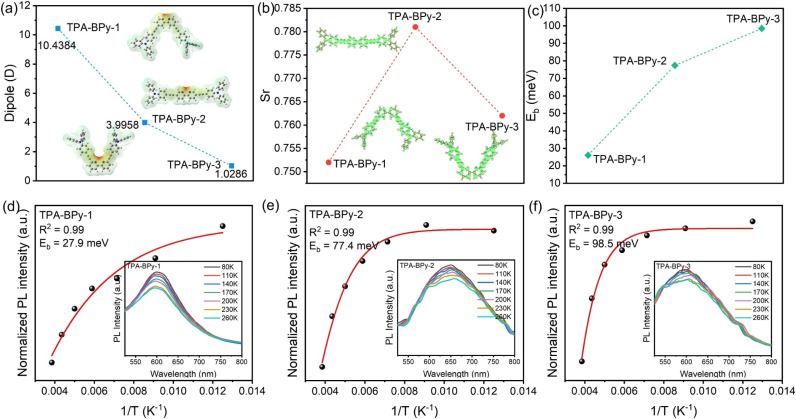
(a) Dipole moments magnitudes and ESP distribution maps of TPA-BPy-1, TPA-BPy-2, and TPA-BPy-3 fragments. (b) The overlap between the electron and hole distributions of three fragments in the S_1_ excited state. The Sr is the hole–electron overlap indicator. (c) Experimental exciton binding energy of TPA-BPy-1, TPA-BPy-2, and TPA-BPy-3 by the Arrhenius equation. (d–f) Temperature-dependent PL spectra and integrated PL emission intensity as a function of temperature from 80 to 260 K.

The TPA-BPy-*n* (*n* = 1, 2, 3) substrates bearing D–π–A units were synthesized by the Sonogashira–Hagihara reaction between tris(4-ethynylphenyl)amine (TPA) and bipyridine skeletons with bromine atoms as the active sites (see ESI† for details). In this process, the bipyridine units as bidentate ligands orient in different crosslinking directions by varying the substituent position of bipyridines, and alkynes are connected to monomers as bridging units and arranged in bonding directions to form a periodic topology. The TPA-BPy-1 and TPA-BPy-3 enforce a V-shaped geometry, promoting twisted polymer chains and non-interpenetrated networks. The TPA-BPy-3 with linear geometry facilitates extended conjugation and rigid, ordered frameworks. Prior to characterizations, the electronic distribution and hole–electron separation efficiency of TPA-BPy-*n* was studied using density functional theory (DFT) calculations for the simplified structure models. These polymer fragments display typical donor–acceptor configurations. The highest occupied molecular orbital (HOMO) and lowest unoccupied molecular orbital (LUMO) are mainly localized on the TPA (electron acceptor) and bipyridine (electron acceptor) unit of the frameworks (Fig. S1†), implying that TPA-BPy-*n* has a strong ICT interaction from donor to acceptor moiety through the π-bridge to reduce the overlap of the hole and electron distributions.^29,30^ Furthermore, electrostatic potential (ESP) distributions of the three model structures show that spatial structural engineering causes the increase of dipole moment and internal spontaneous polarization, resulting in an uneven spatial charge distribution and the formation of localized dipoles within the molecule.^31^ The dipole moment magnitudes of TPA-BPy-1, TPA-BPy-2, and TPA-BPy-3 are 10.4384 D, 3.9958 D, and 1.0286 D, respectively (Fig. 2a). Particularly, the dipole moment of TPA-BPy-1 is significantly higher than that of TPA-BPy-2 and TPA-BPy-3. This elevated molecular dipole moment facilitates efficient charge transfer within the molecule. Theoretical analyses of frontier orbital distributions and molecular dipole moments suggest that the spatial engineering in TPA-BPy-*n* induces localized polarization, enhancing exciton dissociation and the generation of photogenerated carriers under visible light.^32–34^

Further insights into the electron–hole distribution in the excited state of TPA-BPy-1, TPA-BPy-2, and TPA-BPy-3 fragments were obtained by time-dependent-density functional theory (TD-DFT) calculation and electron–hole excitation analysis with *Multiwfn* software.^35–37^ As shown in Fig. S2–S4,† the degree of electron–hole separation of TPA-BPy-1 is remarkably higher than that of TPA-BPy-2 and TPA-BPy-3, where the positive and negative charges are mainly located in the TPA and bipyridine units, respectively. Moreover, some key parameters were calculated to investigate the subtle changes in these fragments during the excitation process (Table S1†). As expected, TPA-BPy-1 has the largest *D* value (1.901 Å) and lowest Sr index (0.752) in the first singlet excited states (S_1_), indicating a better hole–electron separation performance of TPA-BPy-1 compared with TPA-BPy-2 and TPA-BPy-3 (Fig. 2b). The electron density difference between the first excited and ground states further supports this superior hole–electron separation efficiency of TPA-BPy-1. Temperature-dependent photoluminescence (PL) spectroscopy was systematically employed to investigate the charge transfer dynamics in the TPA-BPy-*n* series. As illustrated in Fig. 2c–f, a distinct temperature-dependent enhancement of integrated PL intensity is observed for all TPA-BPy-*n*, following a characteristic pattern of thermally activated nonradiative recombination processes.^38^ This temperature-dependent behavior is quantitatively analyzed through Arrhenius formalism using the equation: *I*(*T*) = *I*_0_/(1 + *A* exp(−*E*_b_/*k*_B_*T*)), where *I*_0_ is the intensity at 0 K, *k*_B_ is the Boltzmann constant, and *E*_b_ is the exciton binding energy.^39^ Through nonlinear least-squares fitting of the experimental data, the exciton binding energies are determined to be 27.9, 77.4, and 98.5 meV for TPA-BPy-1, TPA-BPy-2, and TPA-BPy-3, respectively. This progressive increase in *E*_b_ values along the series (TPA-BPy-1 < TPA-BPy-2 < TPA-BPy-3) establishes a clear correlation between molecular structure and exciton stability. The relatively lower binding energy observed in TPA-BPy-1 suggests enhanced exciton dissociation efficiency compared to its higher counterparts, potentially originating from reduced coulombic interactions.^40^ Theoretical and experimental results collectively show that spatial structural engineering significantly enhances hole–electron separation efficiency in these polymers. The high dipole polarization is beneficial for boosting exciton dissociation efficiency, which plays a vital role in determining their photocatalytic activity.

**Fig. 3:**
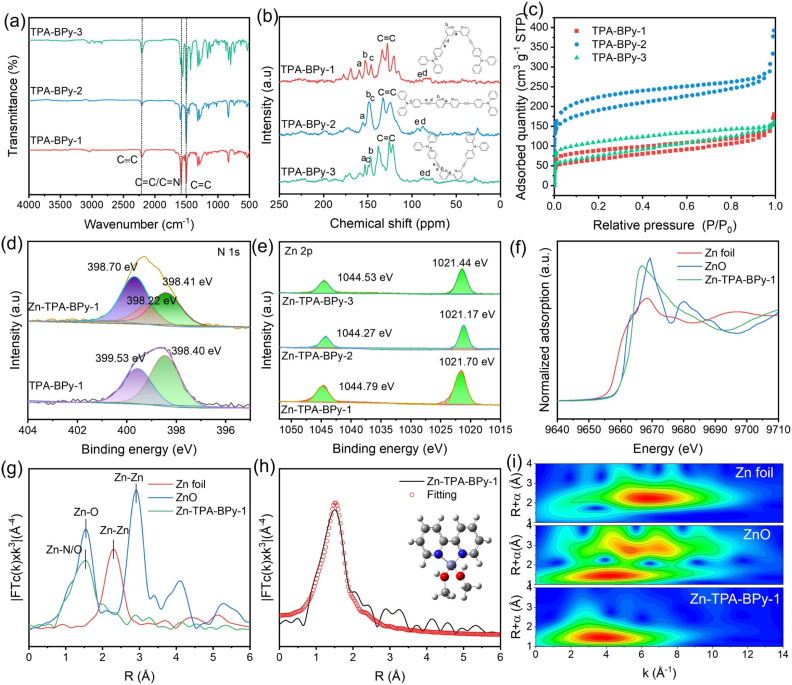
(a) FT-IR spectra, (b) solid-state ^13^C NMR spectra, and (c) N_2_ sorption isotherms of TPA-BPy-*n*. (d) High-resolution N 1s XPS spectra of Zn-TPA-BPy-1 and TPA-BPy-1. (e) Zn 2p XPS spectra of Zn-TPA-BPy-*n*. (f) Zn K-edge XANES and (g) Fourier-transformed EXAFS spectra of Zn foil, ZnO, and Zn-TPA-BPy-1. (h) Zn K-edge XANE fitting analyses for Zn-TPA-BPy-1 in *R* space. (i) The wavelet transforms of EXAFS spectra for Zn-TPA-BPy-1 and reference samples.

The defined chemical structures of TPA-BPy-*n* were systematically confirmed by Fourier transform infrared spectroscopy (FT-IR) and solid-state ^13^C cross-polarization/magic angle spinning nuclear magnetic resonance (CP/MAS NMR) analyses. FT-IR spectra of three CMPs (Fig. 3a) exhibit distinct stretching vibrations at the stretching vibration peak of C≡C at ∼2184 cm^−1^, and the C=C of benzenes at ∼1500 cm^−1^ can be observed (Fig. 3a). Notably, the C=N stretching frequency of pyridine moieties in TPA-BPy-1 appears at ∼1580 cm^−1^ in TPA-BPy-1, displaying obvious shifts compared to TPA-BPy-2 and TPA-BPy-3 due to electron-withdrawing conjugation effects between pyridinic nitrogen and adjacent alkyne groups in the regioisomeric topology. Meanwhile, the C≡C–H and C–Br stretching vibration signals cannot be identified in the FT-IR spectra of CMPs (Fig. S5–S7†), indicating the successful polymerization of monomers. The expected peaks for the carbon atoms in the C≡C bond at ∼87 and ∼90 ppm can be observed in the ^13^C NMR spectra of CMPs (Fig. 3b). The carbon atoms near pyridine N correspond to the peak at about ∼150 ppm, and the peaks of aromatic carbons are found at 116–134 ppm. The PXRD analysis suggests the amorphous nature with a broad hump at 2*θ* = 21.2° due to the interlayer π–π interaction of the phenyl ring (Fig. S8†). In addition, thermogravimetric analysis of TPA-BPy-*n* shows their decomposition temperature above 420 °C (Fig. S9†), demonstrating their good thermal stability. The permanent porosities of TPA-BPy-*n* were investigated by N_2_ adsorption–desorption measurement at 77 K (Fig. 3c). All the sorption isotherms of TPA-BPy-*n* can be identified as the typical type-IV sorption curves with steep gas uptake in the low relative pressure range and distinct hysteresis loop, indicative of microporous and mesoporous characteristics. The Brunauer–Emmett–Teller (BET) specific surface areas of TPA-BPy-1, TPA-BPy-2, and TPA-BPy-3 are calculated to be 237.8, 423.2, and 222.8 m^2^ g^−1^, respectively. Additionally, the pore size distribution curves exhibit that TPA-BPy-1 and TPA-BPy-3 with distorted V-shaped geometry induces tighter packing and balances micro/mesoporosity, yielding intermediate pore size. In contrast, TPA-BPy-2 with uniform geometry and channels can obviously enhance the surface area with main microporosity (Fig. S10†). Scanning electron microscopy (SEM) and transmission electron microscopy (TEM) display that TPA-BPy-*n* possesses a similar particle-stacked morphology with particle sizes in the range of 200–400 nm (Fig. S11†).

Subsequently, to study the influence of the bipyridine ligand's spatial location, Zn species (Zn(NO_3_)_2_) were integrated into dipyridine units through post-modification using methanol solvent (see details in ESI†). The resulting Zn–N bonds restrict C–C bond rotation, enhancing molecular rigidity. Inductively coupled plasma-mass spectrometry (ICP-OES) detection reveals that the Zn contents of Zn-TPA-BPy-1, Zn-TPA-BPy-2, and Zn-TPA-BPy-3 are 1.49, 1.47, and 1.55 wt%, respectively, where only partially bipyridine units are coordinated with Zn^2+^ in the skeletons. FT-IR and PXRD characterizations all reveal that the morphology and structure of TPA-BPy-*n* are retained after incorporating Zn species (Fig. S12 and S13†). Following, take Zn-TPA-BPy-1 for example, N_2_ adsorption–desorption measurement was conducted to investigate the change of porosity and morphology. The N_2_ sorption curve of Zn-TPA-BPy-1 still presents the type IV isotherms with a BET surface area of 202.30 m^2^ g^−1^ (Fig. S14†). The slight decrease in specific surface area can be caused by the space occupations by introducing Zn species. TEM images in Fig. S15† reveal that after incorporating Zn^2+^ ions, the original morphology of TPA-BPy-1 is not changed. Energy dispersive spectroscopy (EDS) elemental mappings of Zn-TPA-BPy-1 distinctly portray the homogeneous spatial distribution of C, N, and Zn, revealing that Zn species are uniformly dispersed across the surface of TPA-BPy-1. X-ray photoelectron spectroscopy (XPS) was conducted to ascertain their chemical compositions and electronic states. The XPS survey spectra confirm the presence of C, N, and target Zn elements in Zn-TPA-BPy-*n* (Fig. S16a†). The high-resolution C 1s spectra are deconvoluted into two main peaks at 284.80 and 285.63 eV, corresponding to C=C/C≡C and C=N bonds, respectively (Fig. S16b†). The high-resolution N 1s spectra of TPA-BPy-1 can be resolved into C–N (398.40 eV) of TPA and C=N (399.53 eV) of bipyridine units.^41^ Compared to the TPA-BPy-1, the C=N signal (398.70 eV) of bipyridine units occur obvious shift in the Zn-TPA-BPy-1, besides, a new Zn–N peak can be deconvolved in the 398.22 eV,^42^ corresponding to the coordination between Zn and bipyridine, as shown in Fig. 3d and S16c.† The Zn 2p spectra exhibit two characteristic Zn 2p_3/2_ peaks (1021.17 eV) and Zn 2p_1/2_ peaks (1044.27 eV) in Zn-TPA-BPy-1 (Fig. 3e), respectively, unambiguously verifying the +2 oxidation state of Zn center.^43^ Moreover, the Zn 2p_3/2_ spectra for Zn-TPA-BPy-1 show distinct binding energy shifts relative to Zn-TPA-BPy-2 and Zn-TPA-BPy-3. The obvious shifts are ascribed to the different coordination environments within the molecular frameworks, which increases the effective nuclear charge experienced by Zn centers through ligand-mediated polarization effects. The Zn K-edge X-ray absorption near edge structure (XANES) spectrum for Zn-TPA-BPy-1 (Fig. 3f) is close to the ZnO, confirming the existence of Zn^2+^ species.^44^ The Zn K-edge extended X-ray absorption fine structure (EXAFS) spectra demonstrate the Zn–N/O coordination with a distance of 1.98 ± 0.02 Å (Zn–N) and 2.09 ± 0.02 Å (Zn–O)^45^ (Fig. 3g). No Zn–Zn bond is observed, implying the isolated Zn atoms anchor on the support. The coordination numbers of the Zn atom are investigated by fitting analysis of EXAFS spectra (Fig. 3h and Table S2†), from which Zn exhibits a coordination number close to 4 (Zn–N_2_O_2_). Specifically, single-atom Zn center is possibly coordinated with bidentate dipyridine and two methanol molecules. The wavelet transforms (WT) analysis of Zn-TPA-BPy-1 further shows an intensity maximum close to ZnO rather than Zn foils (Fig. 3i), suggesting the maintenance of Zn–N/O structure in Zn-TPA-BPy-1.

### Photocatalytic CO_2_ reduction performance of catalysts

**Fig. 4:**
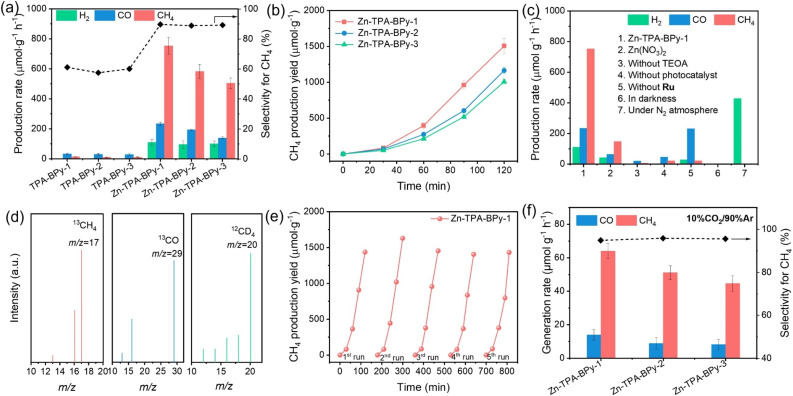
(a) Photocatalytic CO_2_ activities of pristine TPA-BPy-*n* and Zn-TPA-BPy-*n*. (b) Time courses of CH_4_ evolution by photocatalytic CO_2_ reduction using Zn-TPA-BPy-*n* photocatalysts for 2 h, with evacuation every 0.5 h. (c) The photocatalytic performance of Zn-TPA-BPy-1 under different conditions. (d) Mass spectra for CO_2_ reduction of Zn-TPA-BPy-1 using ^13^CO_2_ as the reacting gas or D_2_O as solvent. (e) Cycling experiments of Zn-TPA-BPy-*n*. (f) Photocatalytic activities of Zn-TPA-BPy-*n* under diluted CO_2_ (10% CO_2_ and 90% Ar) atmosphere.

The photocatalytic CO_2_ reduction performance of these samples was investigated under simulated solar illumination conditions in a pure-water system with triethanolamine (TEOA) as electron donor and [Ru(bpy)_3_]Cl_2_·6H_2_O (abbreviated as Ru) as photosensitizer. To verify the role of Zn species in the catalytic reaction, pristine TPA-BPy-*n* was tested. The results show that TPA-BPy-*n* mainly implements CO_2_ reduction to CO with minimal selectivity towards CH_4_ (Fig. 4a). In contrast, the structural incorporation of Zn sites induces a product selectivity shift from predominant CO to CH_4_ evolution, while demonstrating superior catalytic activity compared to transition metal counterparts (*e.g.*, Co, Ni, Cu) (Fig. S17†). Specifically, in terms of the CO_2_-to-CH_4_ conversion, Zn-TPA-BPy-1 achieves a CH_4_ evolution rate of 753.2 μmol g^−1^ h^−1^, nearly 58.9-fold enhancement of TPA-BPy-1, while the selectivity also incredibly increases from 61.1% to 89.7% (electron selectivity). As observed from the time–yield plots, the rate of product evolution increases linearly during the catalytic process (Fig. 4b). The CH_4_ production rate is quite competitive with many reported photocatalysts (Table S3†). Impressively, Zn-TPA-BPy-1 exhibits superior catalytic activity compared to Zn-TPA-BPy-2 and Zn-TPA-BPy-3, which indicates the critical role of metal coordination geometry in modulating active site electronic states. Notably, compared to Zn-TPA-BPy-2 and Zn-TPA-BPy-3, Zn-TPA-BPy-1 still presents higher activity from time–yield plots. Furthermore, the measured apparent quantum yield (AQY) of CH_4_ evolution for Zn-TPA-BPy-1 can achieve the maximum value of 3.45% at 450 nm (Fig. S18†).

Control experiments were conducted to understand the reaction progress, and the results are shown in Fig. 4c. While Zn(NO_3_)_2_ demonstrated catalytic activity for CO_2_ reduction to CH_4_ (146.9 μmol g^−1^ h^−1^), its performance was significantly lower than Zn-TPA-BPy-1, confirming that the TPA-BPy-1 framework is essential for enabling Zn species to selectively drive CH_4_ production. Only extremely small amounts of carbon products could be detected without adding photocatalyst and TEOA, where the carbon products possibly origin from the catalysis of Ru for CO_2_ photoreduction. These results confirm that photocatalyst and TEOA are indispensable in this system. However, in the absence of Ru, Zn-TPA-BPy-1 exhibits predominant CO evolution with a rate of 230.27 μmol g^−1^ h^−1^, accompanied by only trace CH_4_ generation. This marked product distribution disparity highlights the critical role of Ru and photocatalysts in synergistically mediating energy transfer to active sites, thereby facilitating proton-coupled electron transfer during intermediate stabilization in the CO_2_ reduction pathway.^46^ Furthermore, no detectable products are observed in the dark or N_2_ feeding gas condition, which affirms that the formed CH_4_ and CO are indeed derived from CO_2_ photoreduction. The isotope labeling experiment was conducted to verify further the origin of carbon products (Fig. 4d). The typical mass spectrum signals of ^13^CH_4_ (*m*/*z* = 17) and ^13^CO (*m*/*z* = 29) can be observed after pumping ^13^CO_2_ as the only carbon source, verifying that the detected carbon products are indeed generated from the CO_2_ photoreduction. Meanwhile, the appearance of the CD_4_ (*m*/*z* = 20) signal using D_2_O instead of H_2_O suggests that H_2_O is the source of hydrogen in CH_4_ rather than TEOA in the CO_2_-to-CH_4_ conversion. Additionally, residual Pd and Cu from Sonogashira–Hagihara coupling are unavoidable and can potentially contribute to CO_2_ reduction as additional metal cocatalysts. ICP-OES analysis confirmed similar residual Pd and Cu levels across all catalysts (Table S4†), yet their activities differed substantially, especially Zn-TPA-BPy-1 exhibited notably higher activity. This indicates that performance differences likely do not originate from residual Pd or Cu. Furthermore, a control catalyst (Zn-TPA-BPy-1-M) was prepared using fivefold higher Pd(PPh_3_)_4_ and CuI, yielding significantly elevated Pd (2.30 wt%) and Cu (0.75 wt%) content. Notably, the production rates of CH_4_ and CO decreased rather than increasing as expected, whereas H_2_ production was enhanced (Fig. S19†). These experiments indicate that the residual Pd and Cu do not significantly participate in the photocatalytic CO_2_ reduction in this work, but their existence is beneficial for H_2_ evolution, resulting in the decreased selectivity. Besides, cycling tests were also carried out on the Zn-TPA-BPy-*n* to reveal their stability. As displayed in Fig. 4e, the activity of CO_2_ photoreduction to CH_4_ presents a negligible decline after five cycles for Zn-TPA-BPy-1. It is worth noting that Zn-TPA-BPy-1 presents a higher CH_4_ evolution rate observed from the time–yield plots than Zn-TPA-BPy-2 and Zn-TPA-BPy-3 during the five cycles (Fig. S20†). Moreover, the SEM and FT-IR of Zn-TPA-BPy-1 show that the morphology and structure of the catalyst remained unchanged (Fig. S21 and S22†), illustrating the excellent durability of our catalysts.

Inspired by the excellent photocatalytic activity of Zn-TPA-BPy-*n* in a pure CO_2_ atmosphere, diluted CO_2_ (10% CO_2_ and 90% Ar) was then used to further investigate their reduction activities (Fig. 4f). Encouragingly, under visible light irradiation, Zn-TPA-BPy-1 still shows a higher CH_4_ evolution rate of 64.05 μmol g^−1^ h^−1^ with a selectivity of 94.9% by using diluted CO_2_ as the source than Zn-TPA-BPy-2 and Zn-TPA-BPy-3. The remarkable activity of Zn-TPA-BPy-1 for CH_4_ production should be attributed to the synergetic contribution of TPA-BPy-1 and Zn sites in enriching diluted CO_2_.

### Exploration of intrinsic catalytic activity enhancement

**Fig. 5:**
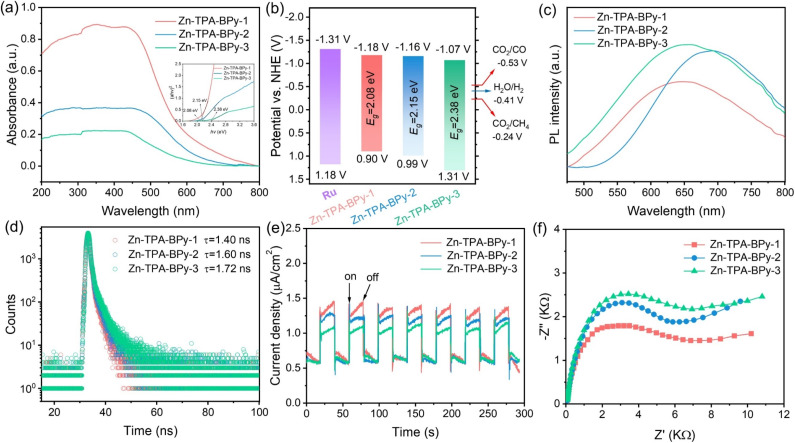
(a) Solid-state UV-vis DRS spectra (inset: Tauc plots), (b) band structure alignment, (c) PL spectra, (d) TR-PL plots, (e) transient photocurrent responses and (f) EIS curves of the as-prepared Zn-TPA-BPy-1, Zn-TPA-BPy-2, and Zn-TPA-BPy-3 samples.

To unveil the underlying reasons for the high CO_2_ reduction activity of Zn-TPA-BPy-1, the mechanistic insights toward the light-harvesting capacity and charge transfer behavior were analyzed. UV-vis diffuse reflectance spectra (DRS) of Zn-TPA-BPy-1 show a higher light absorption intensity and a remarkable red shift of the absorption edge with an expanded absorption tail compared to those of Zn-TPA-BPy-2 and Zn-TPA-BPy-3 (Fig. 5a), indicating the improved optical properties and enhanced light harvesting ability due to distinct configuration. Accordingly, the band gaps (*E*_g_) of these samples are calculated to be 2.08, 2.15, and 2.38 eV for Zn-TPA-BPy-1, Zn-TPA-BPy-2, and Zn-TPA-BPy-3, respectively, from the corresponding Tauc plots. Zn-TPA-BPy-1 exhibits a narrower bandgap, which facilitates the transition of photogenerated electrons.^47^ Mott–Schottky measurements were then carried out to determine the flat band potentials (*E*_FB_) (Fig. S23†). The positive slopes of the M–S plots indicate that Zn-TPA-BPy-*n* are typical n-type semiconductors, and the *E*_FB_ of Zn-TPA-BPy-1, Zn-TPA-BPy-2, and Zn-TPA-BPy-3 can be determined to be −1.18, −1.16, and −1.07 V *vs.* NHE (normal hydrogen electrode, pH = 7), that is, −1.38, −1.36, and −1.27 V (*vs.* Ag/AgCl), respectively. In general, conduction band potential (*E*_CB_) is approximately equal to the *E*_FB_ for n-type semiconductors,^48^ thus valence band potential (*E*_VB_) of Zn-TPA-BPy-*n* series can be calculated to be 0.90, 0.99, and 1.31 V *vs.* NHE, respectively, by the equation *E*_VB_ = *E*_CB_ + *E*_g_. Band alignment in Fig. 5b reveal that all Zn-TPA-BPy-*n* catalysts exhibit more positive *E*_CB_ potentials compared to Ru photosensitizer, creating a substantial thermodynamic driving force for directional electron transfer from photoexcited Ru to Zn catalytic centers. This charge transfer mechanism effectively suppresses electron–hole recombination while maintaining strong redox capacities.^49^ Crucially, *E*_CB_ of Zn-TPA-BPy-*n* has more negative theoretical potentials for CO_2_ reduction to CO and CH_4_.^50^ Such favorable band energetics not only ensure thermodynamic feasibility but also enable kinetically favorable multi-electron transfer processes for CH_4_ production.

To elucidate the photoinduced charge carrier dynamics, we conducted systematic photophysical characterization through steady-state photoluminescence (PL) and time-resolved transient photoluminescence (TRPL) spectroscopy. As shown in Fig. 5c, Zn-TPA-BPy-1 displays marked PL quenching compared with Zn-TPA-BPy-3 and Zn-TPA-BPy-2, indicating that the carrier recombination of Zn-TPA-BPy-1 is greatly suppressed, which represents the higher separation efficiency of photogenerated charges. The TRPL curve of Zn-TPA-BPy-1 in Fig. 5d exhibits that the average carrier lifetime of Zn-TPA-BPy-1 (1.40 ns) is shortened compared to Zn-TPA-BPy-2 (1.60 ns) and Zn-TPA-BPy-3 (1.72 ns), indicating that the photogenerated charges in Zn-TPA-BPy-1 transport much faster. It can be thus speculated that the localized asymmetric charge polarization in TPA-BPy-1 facilitates ultrafast photoinduced charge transfer and charge separation from donor to acceptor, and then rapid electron injection into Zn sites.^51–53^ The transient photocurrent curves and electrochemical impedance spectroscopy (EIS) spectra provided additional corroboration to this observation. Owing to the synergetic effects of enhanced light absorption and inhibited exciton recombination, Zn-TPA-BPy-1 displays higher photocurrent density and smaller semicircle in the Nyquist plots compared to Zn-TPA-BPy-2 and Zn-TPA-BPy-3 (Fig. 5e and f), demonstrating its more photogenerated free charge carriers and efficient interface transfer resistance for improved charge transfer efficiency. Additionally, lifetime, photoconductivity, and resistivity of parent TPA-BPy-1, TPA-BPy-2, and TPA-BPy-3 follows a similar trend; however, their efficiency was significantly improved upon Zn incorporation (Fig. S24–S26†). Moreover, TD-DFT hole–electron analysis reveals electron/hole distribution in bipyridine units and Zn sites. Zn-TPA-BPy-*n* exhibits lower Sr and higher *D* values than TPA-BPy-*n* (Fig. S27 and Table S5†), indicating Zn sites can also facilitate charge transfer and charge separation. It can be thus concluded that the regioisomer-dependent π-topology significantly influences polarity and optoelectronic properties. Crucially, asymmetric structures with high dipole polarization enable effective charge transport and separation, directing charges to bipyridine units (electron collection center) to activate the Zn site for CO_2_ reduction.

### Underlying mechanism of photocatalytic CO_2_ reduction

**Fig. 6:**
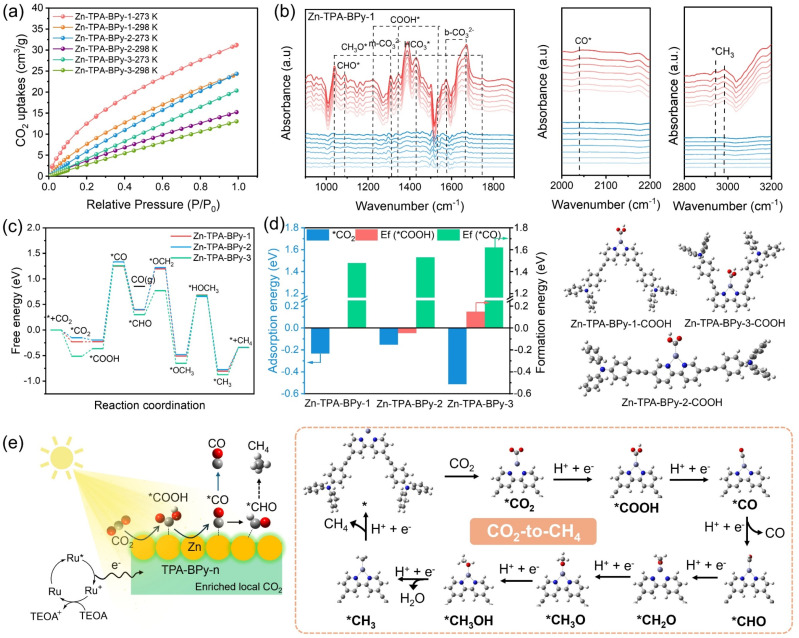
(a) CO_2_ adsorption isotherms of Zn-TPA-BPy-1, Zn-TPA-BPy-2, and Zn-TPA-BPy-3 samples. (b) *In situ* DRIFTS spectra of Zn-TPA-BPy-1 sample showing the reaction pathways of photocatalytic CO_2_-to-CH_4_ process in the dark (blue lines) and light irradiation (red lines). (c) Gibbs free energy (Δ*G*, eV) profiles of photoreduction of CO_2_ over Zn-TPA-BPy-*n*. (d) The adsorption energy of *CO_2_ and formation energy of *COOH and *CO on Zn-TPA-BPy-*n*. (e) Schematic illustration showing possible reaction pathway for photocatalytic CO_2_ reduction on the Zn-TPA-BPy-*n*.

To unveil the actual reaction mechanism, the CO_2_ adsorption capacities of Zn-TPA-BPy-*n* were first evaluated. The CO_2_ adsorption isotherms in Fig. 6a illustrate that all Zn-TPA-BPy-*n* possesses high CO_2_ adsorption capacities. These results suggest that TPA-BPy-*n* can enrich local CO_2_ molecules on the catalyst surface, and the high CO_2_ physisorption is beneficial for promoting the CO_2_ reduction process. Notably, Zn-TPA-BPy-1 exhibits a higher CO_2_ uptake than Zn-TPA-BPy-2 and Zn-TPA-BPy-3. This physical adsorption is likely to be enhanced by the upgraded spatial configuration of TPA-BPy-1, which improves its capability to activate CO_2_ molecules. The *in situ* diffuse reflectance infrared Fourier transformation spectroscopy (DRIFTS) measurements were conducted on Zn-TPA-BPy-1 with light irradiation to probe the key reaction intermediates (Fig. 6b). When CO_2_ molecules are injected into the reactor under a dark environment, several peaks assigned to bidentate carbonate (b-CO_3_^2−^, 1575 and 1673 cm^−1^), monotonic carbonate (m-CO_3_^2−^, 1310 cm^−1^), and bicarbonate (HCO_3_^−^, 1431 cm^−1^) species^54,55^ are observed in the collected spectra, indicating that CO_2_ molecules are adsorbed and activated on the surface. Upon illumination, these peak intensities are enhanced, while new absorption peaks of *COOH (1224, 1343, and 1532 cm^−1^)^56,57^ and *CO absorption intermediates (1917 cm^−1^)^58^ gradually appear. More importantly, the pivotal intermediate *CHO (1088 cm^−1^) for the formation of CH_4_ is observed,^59^ which can be ascribed to the proton from the vapor reacted with *CO intermediate. Moreover, the characteristic peaks of *CH_3_O (974, 1040, and 1749 cm^−1^) and *CH_3_ (2891 and 2982 cm^−1^) also appear, representing key intermediates for the generation of CH_4_. This reaction pathway leads to photocatalytic CO_2_-to-CH_4_ reaction pathways of the Zn-TPA-BPy-1 sample under illumination.

DFT calculations were then conducted on Zn-TPA-BPy-*n* to further verify the possible photocatalytic CO_2_-to-CH_4_ pathway. According to the free energy diagrams (Fig. 6c and d), the formations of *CO_2_ on Zn-TPA-BPy-*n* are exothermic processes, indicating the high efficiency of CO_2_ activation due to the synergistic effect between the TPA-BPy-*n* and Zn site. Despite lower adsorption energy, Zn-TPA-BPy-3 requires 0.41 eV energy expenditure for the *COOH formation step, higher than Zn-TPA-BPy-1 and Zn-TPA-BPy-2. Note that the C–O bond cleavage in *COOH to form *CO is a highly endergonic, rate-limiting step. Zn-TPA-BPy-1 exhibits a lower energy barrier for *CO formation (Δ*G* = 1.48 eV) than Zn-TPA-BPy-2 (1.53 eV) and Zn-TPA-BPy-3 (1.62 eV). By balancing *CO_2_ adsorption energy with *COOH and *CO formation energies, TPA-BPy-1 lowers reaction barriers, strengthens metal-site bonding, and enhances photocatalytic CO_2_ reduction activity. Moreover, *CO desorption and hydrogenation to *CHO, the key intermediate to form CH_4_, are exothermic spontaneously. However, the *CHO intermediate is validated to form preferentially during the *CO transformation process, thereby diminishing the selectivity of CO and realizing the selective CH_4_ evolution. Additionally, the formation of CH_3_OH* intermediates can be another rate-limiting step for hydrogenation processes, where Zn-TPA-BPy-3 exhibits a higher energy barrier compared to Zn-TPA-BPy-1 and Zn-TPA-BPy-2, impeding *CH_3_ formation from CH_3_OH* *via* dehydration. These DFT calculations suggest that the whole process of CO_2_ photoreduction into CH_4_ reaction on Zn-TPA-BPy-1 is more favorable in thermodynamics. Thus, it can be believed that the high polarity and outside-channel active sites are beneficial for modulating the reaction energy barrier of *COOH and *CO intermediates to facilitate the selectivity-determining protonation of *CO to *CHO intermediates, thereby regulating the reaction activity during the CO_2_ reduction process.

Based on the above results, a reasonable mechanism for CO_2_ photoreduction on Zn-TPA-BPy-*n* was proposed, as illustrated in Fig. 6e. Under visible light irradiation, photoexcited electrons are generated on the Ru photosensitizer, which then transfer to TPA-BPy-*n* and flow to the Zn sites to participate in the reduction of CO_2_ molecules adsorbed on the surface of catalysts, during which TEOA acted as a sacrificial agent to complete the Ru cycle.^60^ We then carried out PL spectroscopy to verify the photogenerated electron transfer process. As shown in Fig. S28,† adding Zn-TPA-BPy-1 quenches the emission of Ru (*λ*_em_ = *ca.* 620 nm), while no significant change occurs when adding TEOA, which provides an evidence that the catalyst effectively takes up the photogenerated electrons from Ru to achieve the eight-electron CH_4_ pathway by continuous electron transport.^61^ The excellent activity and selectivity of Zn-TPA-BPy-1 for photocatalytic CO_2_ reduction to CH_4_ primarily stem from its enhanced charge transfer ability, lower activation barriers for the generation of the key intermediates *COOH and *CO, and the thermodynamically favorable formation of *CHO. These factors are closely related to the unique spatial configuration, endowing Zn atoms with a higher activity.

## Conclusions

In summary, three Zn-TPA-BPy-*n* (*n* = 1, 2, 3) catalysts are constructed by varying the arrangement in bonding directions of bipyridine substituents to form spatially distinct periodic topologies for photocatalytic CO_2_ reduction. Remarkably high catalytic activities are exhibited by the designed systems, particularly Zn-TPA-BPy-1, which achieves a CH_4_ production rate of 753.2 μmol g^−1^ h^−1^ with 89.7% selectivity. Experimental and theoretical results reveal that TPA-BPy-1 with high dipole moment and sites anchored outside pore walls can facilitate dipole-induced internal polarization and mass transport, enabling the Zn active site for efficient CO_2_ conversion. Detailed mechanistic studies demonstrate that the synergistic effect of the Zn sites and TPA-BPy-1 reduces the energy barrier for *COOH and *CO intermediates, and thermodynamically favors the formation of *CHO intermediates, thus boosting selective photoreduction of CO_2_ to CH_4_. This work provides new insight into designing efficient organic photocatalysts containing more abundant metals toward highly selective CH_4_ production and a reasonable mechanism for CO_2_ reduction.

## Author contributions

X. L. and Y. C. supervised the project. J. W. and L. C. synthesized the catalysts. J. W. and L. C. carried out photocatalytic experiments. J. W., L. C., and H. X. characterized the materials. T. Z. carried out synchrotron radiation. X. L. conducted DFT calculations. X. L. and Y. C. co-wrote the manuscript. All authors discussed the results and contributed to the preparation of the manuscript.

## Conflicts of interest

The authors declare no conflict of interest.

## Supplementary Material

SC-016-D5SC02835C-s001

## Data Availability

Further details of the experimental procedure, figures, tables, and calculations are available in the ESI.†
