# Supplementary material for: Spatially programmed regioisomeric conjugated microporous polymers modulating zinc sites for selective CO2 photoreduction to CH4
Source: Chem Sci. 2025 Jun 30;16(30):13893–904. doi: 10.1039/d5sc02835c (PMC12226928; doi:10.1039/d5sc02835c)
Supplement: SC-016-D5SC02835C-s001 [file SC-016-D5SC02835C-s001.pdf]

Supplementary Information (SI) for Chemical Science.
This journal is © The Royal Society of Chemistry 2025

Supporting Information

Spatially Programmed Regioisomeric Conjugated Microporous Polymers Modulating Zinc Sites for Selective CO_2 Photoreduction to CH_4

Xingwang Lan,[a] Juan Wang,[a] Lu Chen[a], Haobo Xu,[a] Tianjun Zhang,[a] Yong Chen[b,c*]*

[a] College of Chemistry and Materials Science, Key Laboratory of Chemical Biology of Hebei Province, Hebei Research Center of the Basic Discipline of Synthetic Chemistry, Institute of Life Science and Green Development, Hebei University, Baoding, Hebei, 071002, P.R. China

E-mail: lanxingwang@hbu.edu.cn (X. Lan)

[b] Key Laboratory of Photochemical Conversion and Optoelectronic Materials & CAS-HKU Joint Laboratory on New Materials, Technical Institute of Physics and Chemistry, Chinese Academy of Sciences, Beijing 100190, P. R. China

E-mail: chenyong@mail.ipc.ac.cn (Y. Chen)

[c] University of Chinese Academy of Sciences, Beijing 100049, P. R. China

Section 1. Experimental

1.1 Materials

All the reagents including trimethylsilylacetylene, 1,3,5-tris(4-bromophenyl) benzene, 4,4'-dibromo-2,2'-bipyridyl, 5,5'-dibromo-2,2'-bipyridyl, 6,6'-dibromo-2,2'- bipyridyl were purchased from LeYan.com. Iron nitrate nonahydrate ($Fe(NO_3)_3 \cdot 9H_2O$), copper nitrate hydrate ($Cu(NO_3)_2 \cdot H_2O$), cobalt nitrate hexahydrate ($Co(NO_3)_2 \cdot 6H_2O$), nickel nitrate hexahydrate ($Ni(NO_3)_2 \cdot 6H_2O$), zinc nitrate hexahydrate ($Zn(NO_3)_2 \cdot 6H_2O$) were purchased from Beijing energy engineering technologies Co., Ltd. $[Ru(bpy)_3]Cl_2 \cdot 6H_2O$, and $Pd(PPh_3)_4$ were purchased from Bide Pharmatech Co., Ltd. Other chemical reagents were purchased from local chemical suppliers. All the commercial chemicals were directly used without further purification.

1.2 Synthesis of TPA-BPy-n (n = 1, 2, 3)

Typically, for the synthesis of TPA-BPy-1, 4,4'-dibromo-2,2'-bipyridine (94.00 mg, 0.30 mmol), tris(4-ethynylphenyl)amine (64.00 mg, 0.20 mmol), CuI (10.00 mg, 0.05 mmol), and $Pd(PPh_3)_4$ (10.00 mg, 0.01 mmol) were charged to a 15 mL Schlenk vessel, and then a mixed solution of 10 mL anhydrous triethylamine (Et_3N) and *N,N*-dimethylformamide (DMF) (volume ratio 1:1) was added. The mixture was sonicated to homogeneous and then degassed by three freeze-pump-thaw cycles. Afterward, the reaction was heated and stirred at 120 °C for 72 h. After completion, the mixture was cooled to room temperature, and the solution was filtered to yield a yellow precipitate. The obtained product was washed sequentially with excess H_2O, THF, CH_3OH, and acetone to remove unreacted reactants. In order to further remove excess monomers and metal catalysts, the powder sample was purified by Soxhlet extraction with methanol and THF for 48 h, followed by vacuum drying at 60 °C overnight. The final product, a yellow powder, was obtained and designated as TPA-BPy-1 with a yield of

83%. The TPA-BPy-2 and TPA-BPy-3 were synthesized similarly to the TPA-BPy-1, except for using 5,5'-dibromo-2,2'-bipyridine (94.00 mg, 0.30 mmol) and 6,6'-dibromo-2,2'-bipyridine (94.00 mg, 0.30 mmol) as reactants, finally obtaining TPA-BPy-2 as an orange-yellow powder with a yield of 85% and TPA-BPy-3 as a yellow powder with a yield of 85%.

1.3 Synthesis of Zn-TPA-BPy-n (n = 1, 2, 3)

The Zn-TPA-BPy-n was prepared *via* a simple post-modification method. In brief, $Zn(NO_3)_2·6H_2O$ (0.2 mmol) as a precursor was added to the TPA-BPy-n (50 mg) in methanol (20 mL) solution. The mixture was agitated at 60 °C under a nitrogen atmosphere for 24 h. After completion, the obtained solid was filtered, washed thoroughly with methanol, and dried at 60 °C under a vacuum overnight to produce Zn-TPA-BPy-n. Similarly, other metal species, *eg.* $Fe(NO_3)_3·9H_2O$, $Co(NO_3)_2·6H_2O$, $Ni(NO_3)_2·6H_2O$, $Cu(NO_3)_2·H_2O$, were employed instead to $Zn(NO_3)_2·6H_2O$ to obtain the M-TPA-BPy-n.

1.4 Photocatalytic CO_2 reduction

The photocatalytic CO_2 reduction experiments were performed in a 100 mL optical reaction vessel with stirring at ambient temperature. First, the photocatalyst (1.0 mg) and $[Ru(bpy)_3]Cl_2·6H_2O$ (4.5 mg) were dispersed into 5 mL of a mixture of triethanolamine, deionized water, and acetonitrile (1:2:2 volume ratio). After homogeneous mixing, the suspension was purged with CO_2 for 10 minutes. The light irradiation came from a PLS-LED 100C white LED lamp (60 W, Beijing Perfect Light, China) with the light intensity of about 471.4 mW/cm^2, measured by a PL-MW2000 Optical Power Meter (Beijing Perfect Light, China). After every 0.5 hours of light irradiation, a gas product (1 mL) was sampled using a gastight syringe and analyzed with a gas chromatograph (GC9790II plus, Fuli Analytical Instrument Co.,

Ltd.). The hydrocarbon products were determined using a flame ionization detector (FID) and a thermal conductivity detector (TCD), respectively. The experimental component and amount of gas products were identified using the standard gas. The possible liquid products were detected by ^{1}H NMR.

The product selectivity of photocatalytic CO_2 reduction to CH_4 was calculated using the following formula, where n represents the production of CO_2 photoreduction products (mmol) after 2 h.

$$CH_4 \text{ selectivity } (\%) = \frac{8n(CH_4)}{8n(CH_4) + 2n(CO) + 2n(H_2)} \times 100\%$$

1.5 Recyclability test

The recycled experiments for photocatalytic CO_2 reduction were performed under the same conditions to evaluate the stability and reusability of the catalyst in a long-term operation. After the reaction was completed, the catalyst was centrifuged and washed using acetonitrile, and then a fresh solution of $[Ru(bpy)_3]Cl_2 \cdot 6H_2O$ as described above was injected to conduct the next cycle.

1.6 The apparent quantum yield (AQY) measurement

The apparent quantum yield (AQY) of CH_4 production was measured under the same photocatalytic reaction condition determined by using monochromatic light sources with wavelengths of 350, 420, 450, 475, 520, and 600 nm for irradiation for 2 h. The AQY value was calculated based on the following equation.

$$AQY\% = \frac{8\,n\,(CH_4)\,N_A\,h\,c}{S\,P\,t\,\lambda} \times 100\%$$

Where, n is the amount of evolved CO (mol), N_A is Avogadro constant (6.022×10^{23} mol^{-1}), h is the Planck constant (6.626×10^{-34} J·s), c is the speed of light (3×10^8 m/s), S is the irradiation area (cm^2), P is the intensity of irradiation light

(W/cm^2), t is the photoreaction time (s), λ is the wavelength of the monochromatic light.

1.7 Characterization

Fourier translation infrared spectra (FT-IR) were collected on a Thermo Scientific Nicolet iS10 instrument. The UV-vis diffuse reflectance spectra of all samples were determined using the Shimadzu UV-3600 near-infrared UV-visible spectrophotometer from SHIMADZU, Japan. Solid-state ^{13}C cross-polarization/magic-angle spinning solid-state nuclear magnetic resonance (CP/MAS ssNMR) spectra were collected on a Bruker AVANCE III HD 400MHz instrument. The thermogravimetric analysis (TGA) was tested using the German type STA449F3 instrument under N_2 atmosphere. The N_2 adsorption-desorption isotherm was analyzed at 77 K using an Autosorb-iQ-MP 3000 analyzer. Prior to testing, samples were degassed for 6 h on an analyzer port at 120°C under vacuum. Surface areas were calculated based on Brunauer-Emmett-Teller (BET) method. Pore size distribution curves were obtained *via* non-local density functional theory (NLDFT) method. Power X-ray diffraction (XRD) was performed on a Bruker D8 ADVANCE instrument with the X-ray source of Cu Kα radiation and data were collected with a scan rate of 0.1 step/s in the range of 5-40°. Scanning electron microscopy (SEM) images were obtained from ZEISS Sigma 300 Emission Electron Microscope. Transmission electron microscopy (TEM) was obtained from JEOL JEM-2100 Field Emission Electron Microscope. Inductively coupled plasma-optical emission spectrometry (ICP-OES) was carried out on an Agilent 5110 instrument. XPS X-ray photoelectron spectra (XPS) data were collected using a Thermo Scientific K-Alpha instrument, which was analyzed by the calibration using the binding energy of 284.8 eV of C 1s. Steady-state photoluminescence (PL) spectra of the samples in solvents and solid powders were recorded at room

temperature on a HITACHI F-7000 spectrophotometer. Time-resolved PL spectra were recorded at Edinburgh FS5 spectrophotometer.

1.8 Photoelectrochemical measurements

All the electrochemical measurements (photocurrent, the Mott–Schottky spots and EIS) were carried out using CHI 760E, CH Instruments Inc., Shanghai. A three-electrode system was used in 0.2 M Na_2SO_4 electrolyte with Ag/AgCl electrode (saturated with KCl) as the reference electrode, Pt wire as the auxiliary electrode and catalyst-coated indium-doped tin oxide (ITO) as the working electrode. The working electrode was prepared by dispersing 2.0 mg of sample in 475 μL of anhydrous ethanol containing 25 μL of Nafion solution for 5 min. 100 μL of the solution was added dropwise onto an ITO glass slide with an effective area of 1 cm2 and dried at room temperature. Mott−Schottky (M−S) plots were recorded at different frequencies of 1500 Hz. Electrochemical impedance measurements were recorded over a 0.01-1000 Hz frequency range. The transient photocurrent responses were recorded over a sampling interval of 20 s using a 60W white LED lamp (PLS-LED 100C, Perfectlight, China).

1.9 In-situ diffuse reflectance infrared Fourier transform spectroscopy (DRIFTS)

In-situ DRIFTS spectra was carried out with a Nicolet iS5 dual-beam spectroscopy. 20 mg of photocatalyst was placed in the *in-situ* reaction cell, and then CO_2 with H_2O vapor was continuously fed into the cell for 30 min to remove air and to reach adsorption-desorption equilibrium at the photocatalyst surface. With the dark state as background, the samples were irradiated with a white LED light source (60 W, PLS-LED 100C, Perfect Light, China) under the moist CO_2 gas flow condition, and the data were collected every 10 minutes.

1.10 Theoretical-calculation details

All computations were studied by density functional theory (DFT), where the related structures were optimized by Gaussian 09 package with Grimme's D3 correction.[1-3] We used a represented fragment rather than the periodic CMP framework, as the description of the charged system is difficult for the periodic system. The optimization calculations were performed with the B3LYP level of theory with SDD basis set for Zn atoms and 6-311G* basis set for the remaining atoms. The implicit solvent effects were considered with the PCM (polarized continuum model) using the water solvent to model a real environment. Harmonic vibrational frequency was performed at the same level to guarantee that there is no imaginary frequency in that molecule. The H^+/e^- pair chemical potential value is half of the H_2 gas phase, whose reference is the reversible hydrogen electrode (RHE).[4] CAM-B3LYP/6-311G** was used for the calculation of excited states. Analysis of electrons and holes during electron excitation was estimated the *Multiwfn* 3.8 software package, and the charge density difference map was also presented by the Multiwfn program.[5]

Section 2. Supplementary Figure and Tables

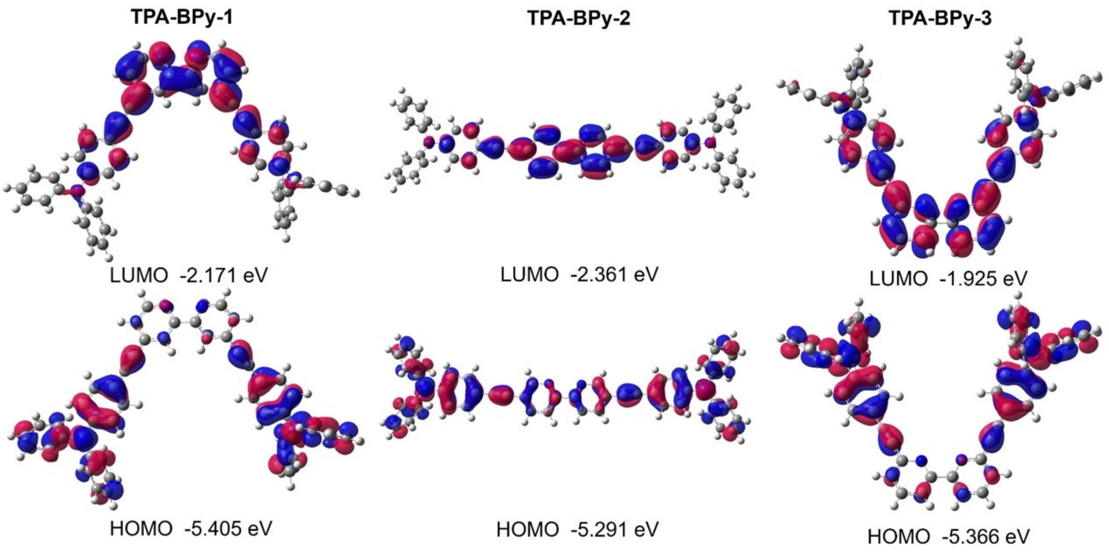

Figure S1. Frontier molecular orbitals of TPA-BPy-1, TPA-BPy-2, and TPA-BPy-3.

Table S1. *D, H,* and *Sr* indices for measuring electron–hole separation

	D (Å)[a]	*H (Å)*[b]	*Sr*[c]
**S1(TPA-BPy-1)**	1.901	6.755	0.752
S2	1.959	6.892	0.745
S3	0.092	5.663	0.728
S4	0.095	5.664	0.728
**S1(TPA-BPy-2)**	0.211	7.664	0.781
S2	0.223	9.100	0.742
S3	0.097	11.170	0.726
S4	0.111	11.170	0.726
**S1(TPA-BPy-3)**	1.605	6.403	0.762

S2	1.715	6.529	0.75395
S3	0.081	7.249	0.72179
S4	0.096	7.259	0.72363

[a]The distance of charge transfer. [b] Average extension of holes and electrons. [c] Overlap between electron and hole distribution.

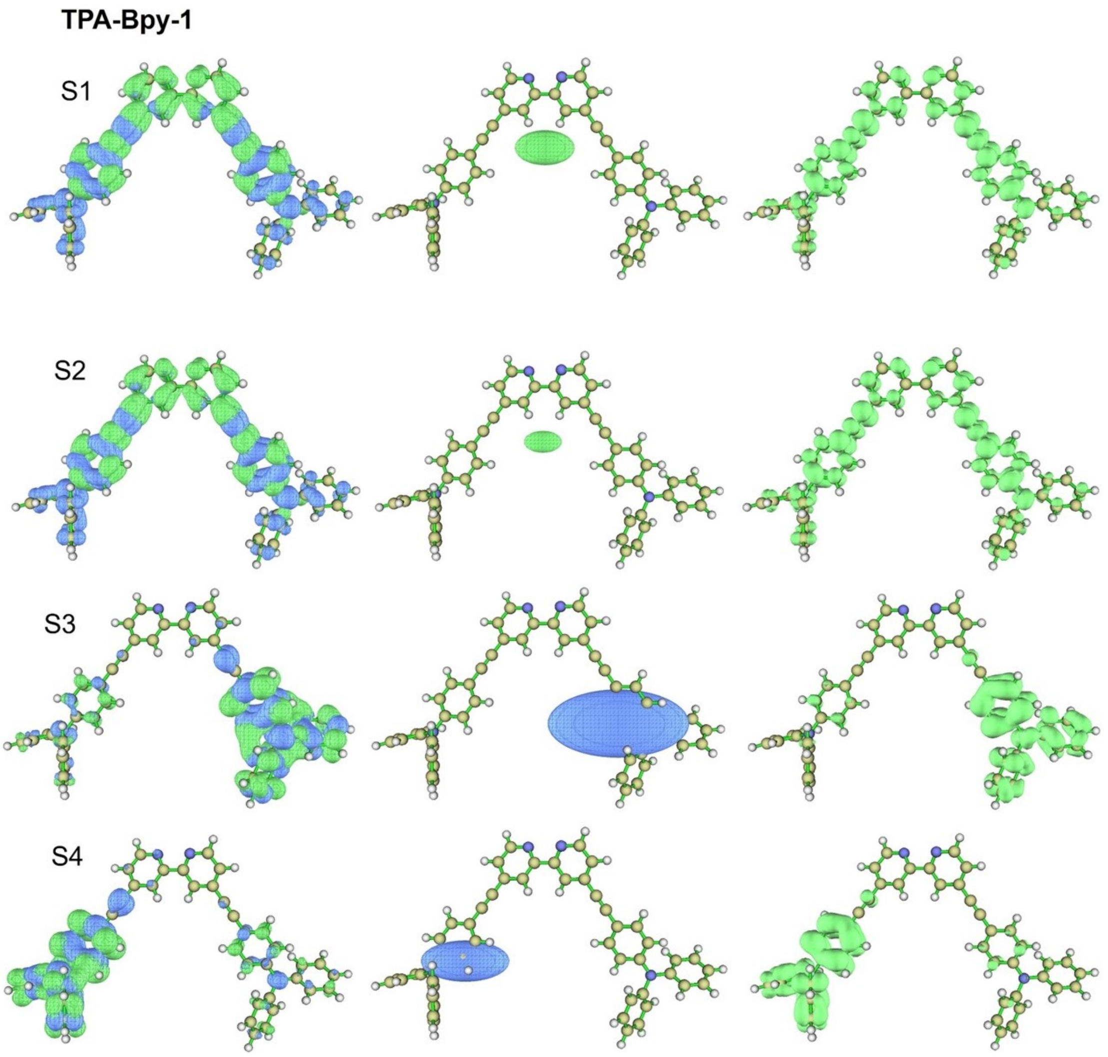

Figure S2. The hole-electron density distributions of S1-S4 excited states of TPA-BPy-1 fragments (isovalue = 0.0005 a.u.). The hole (blue) and electron (green) distribution in the S1-S4 excited states (First column). The smoothing description of hole and electron distribution (Second column). The overlap between electron and

hole distribution of polymer fragments (Third column).

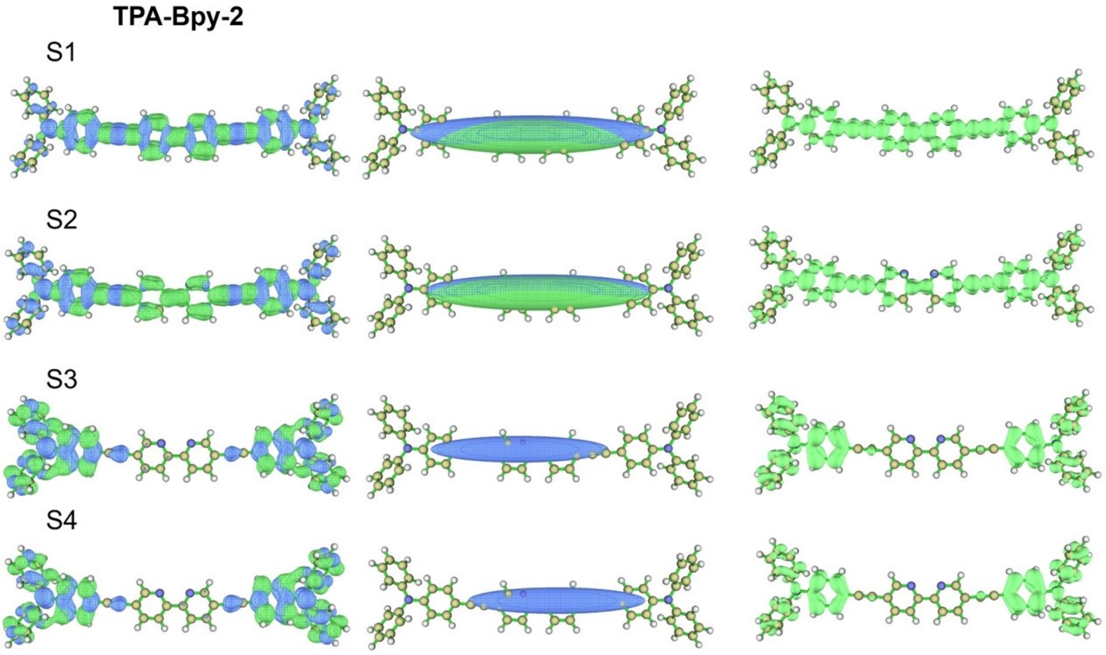

Figure S3. The hole-electron density distributions of S1-S4 excited states of TPA-BPy-2 fragments (isovalue = 0.0005 a.u.). The hole (blue) and electron (green) distribution in the S1-S4 excited states (First column). The smoothing description of hole and electron distribution (Second column). The overlap between electron and hole distribution of polymer fragments (Third column).

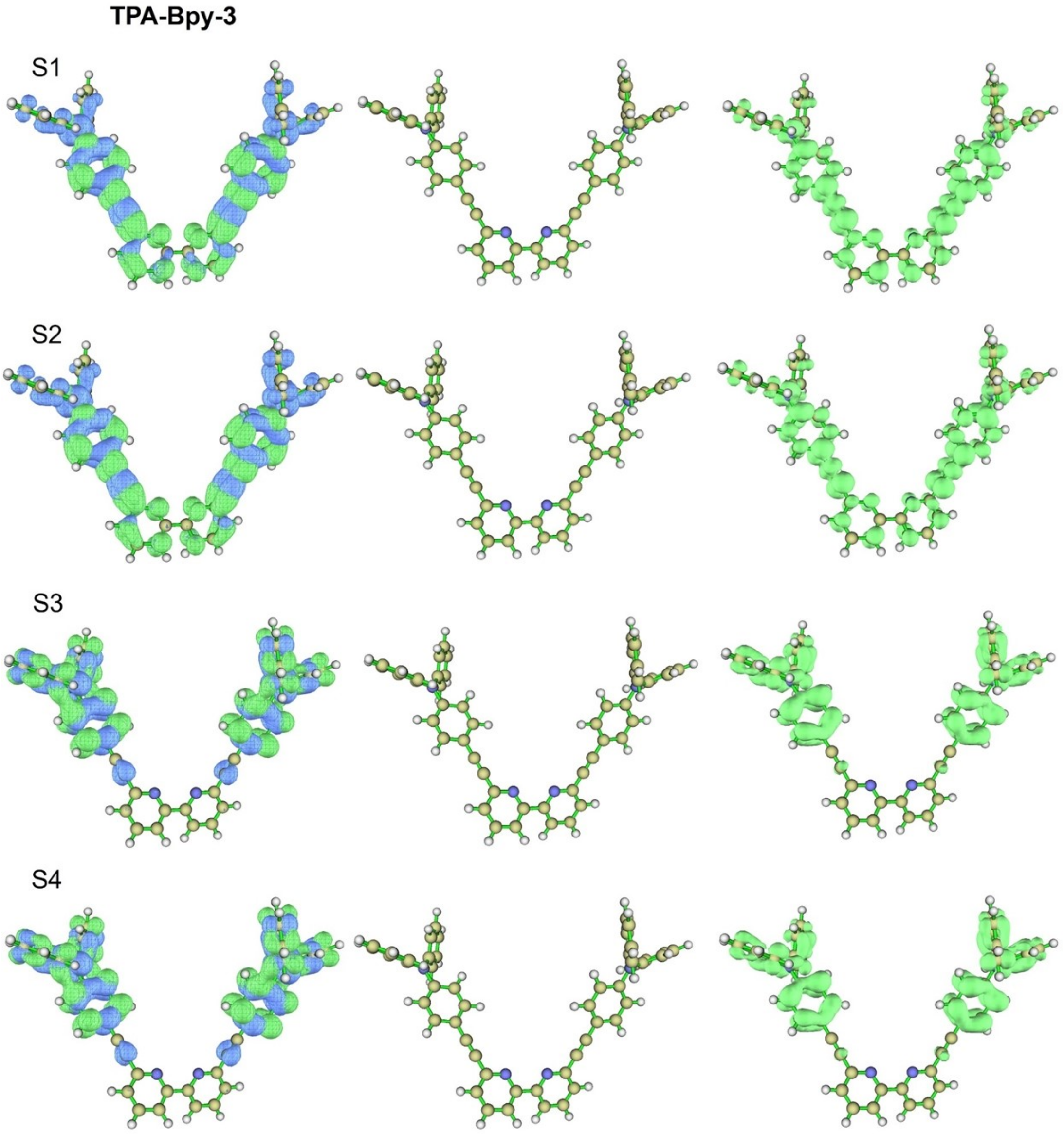

Figure S4. The hole-electron density distributions of S1-S4 excited states of TPA-BPy-2 fragments (isovalue = 0.0005 a.u.). The hole (blue) and electron (green) distribution in the S1-S4 excited states (First column). The smoothing description of hole and electron distribution (Second column). The overlap between electron and hole distribution of polymer fragments (Third column).

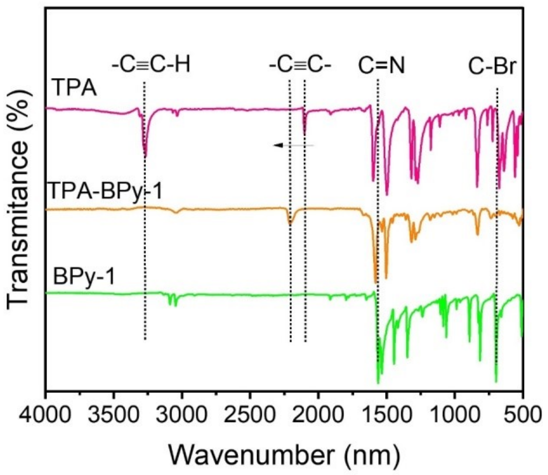

Figure S5. FT-IR spectra of TPA-BPy-1, TPA, and BPy-1.

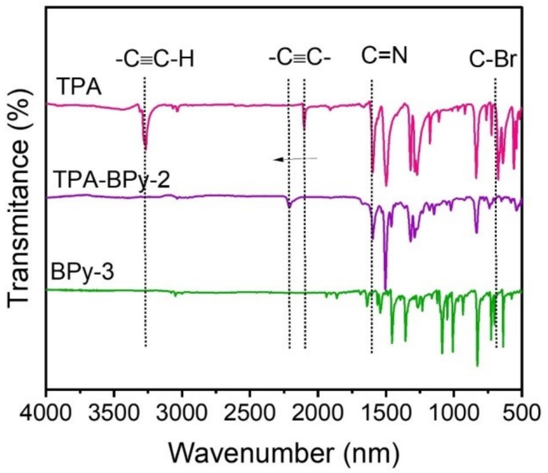

Figure S6. FT-IR spectra of TPA-BPy-2, TPA, and BPy-2.

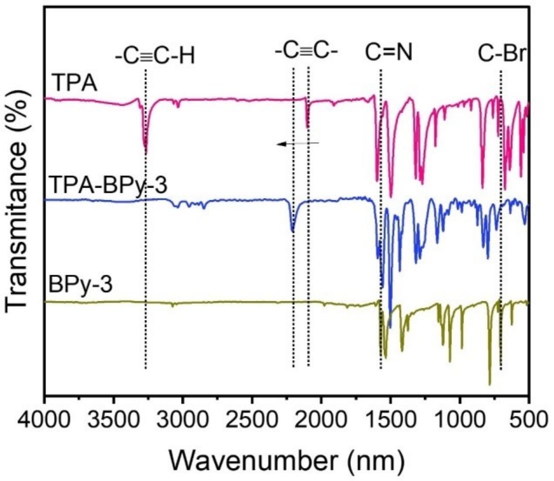

Figure S7. FT-IR spectra of TPA-BPy-3, TPA, and BPy-3.

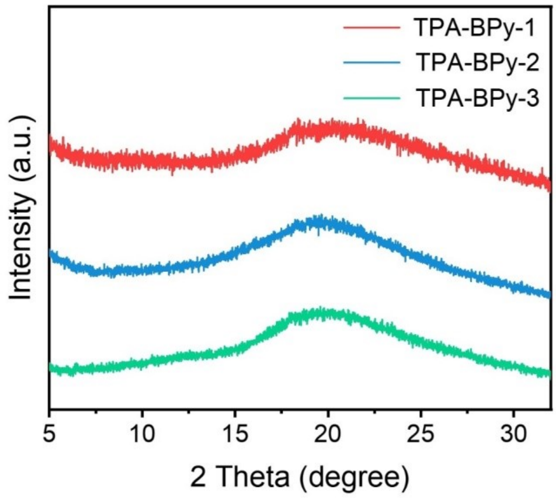

Figure S8. PXRD patterns of TPA-BPy-1, TPA-BPy-2, and TPA-BPy-3.

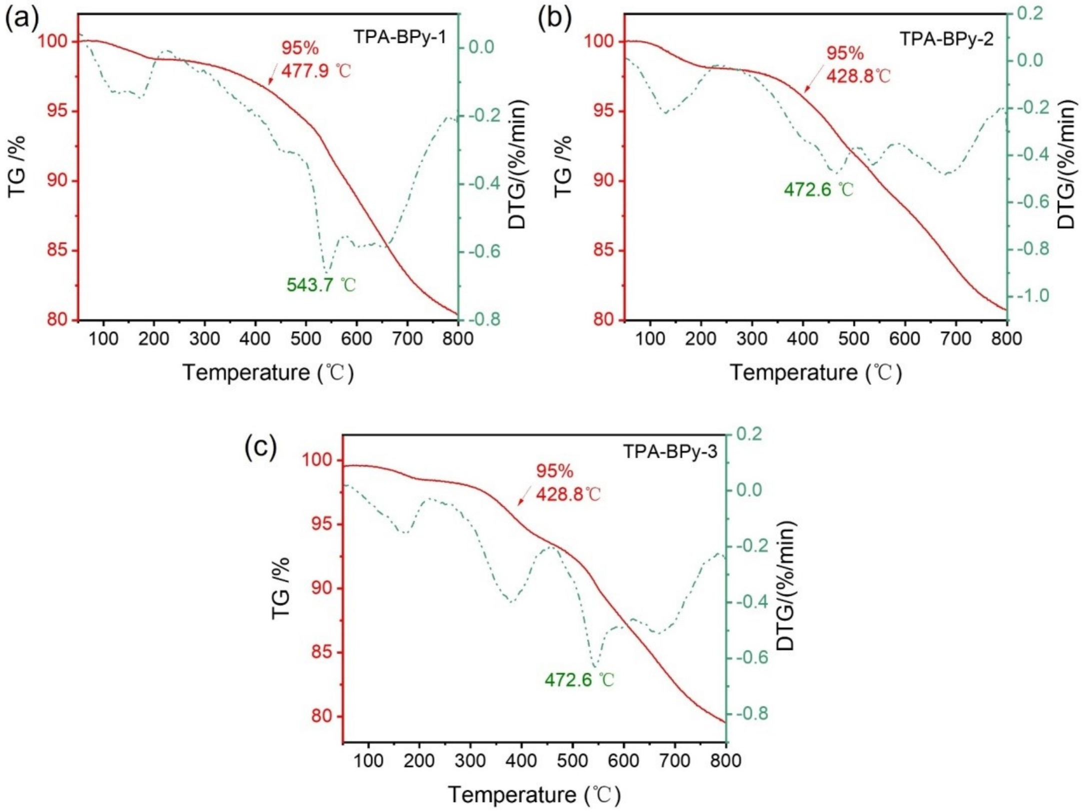

Figure S9. Thermogravimetric analyses of TPA-BPy-1, TPA-BPy-2, and TPA-BPy-3.

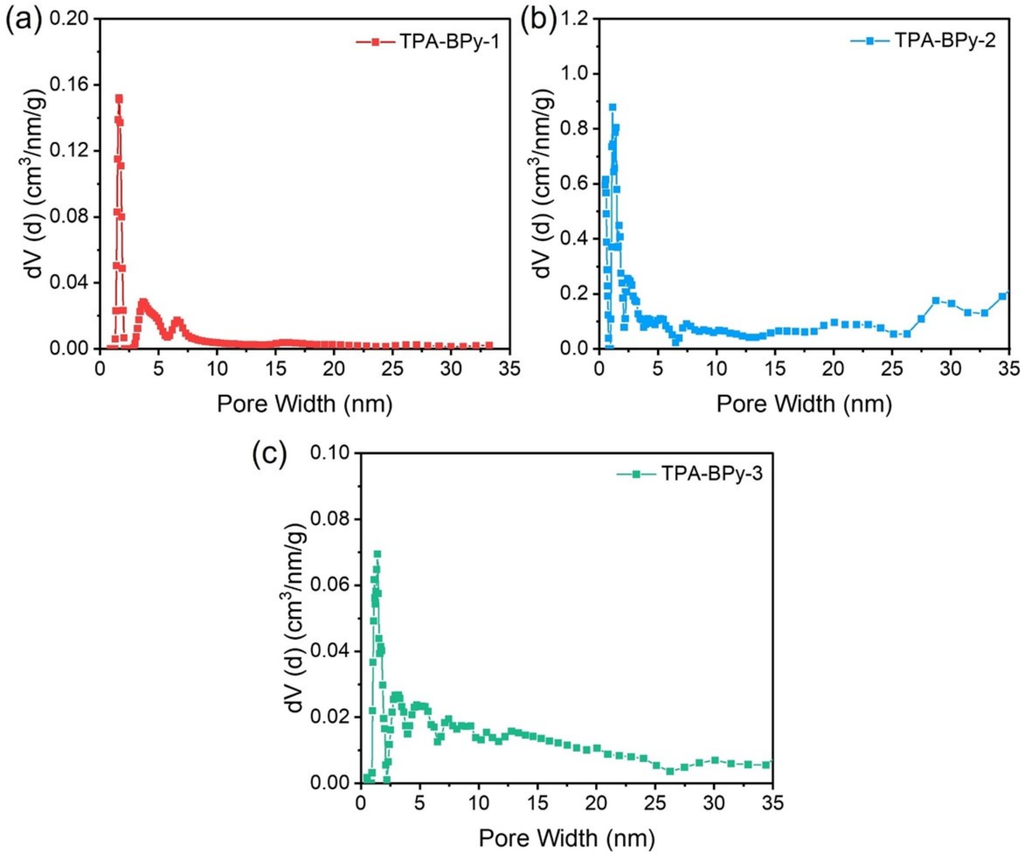

Figure S10. Pore size distributions of TPA-BPy-1, TPA-BPy-2, and TPA-BPy-3.

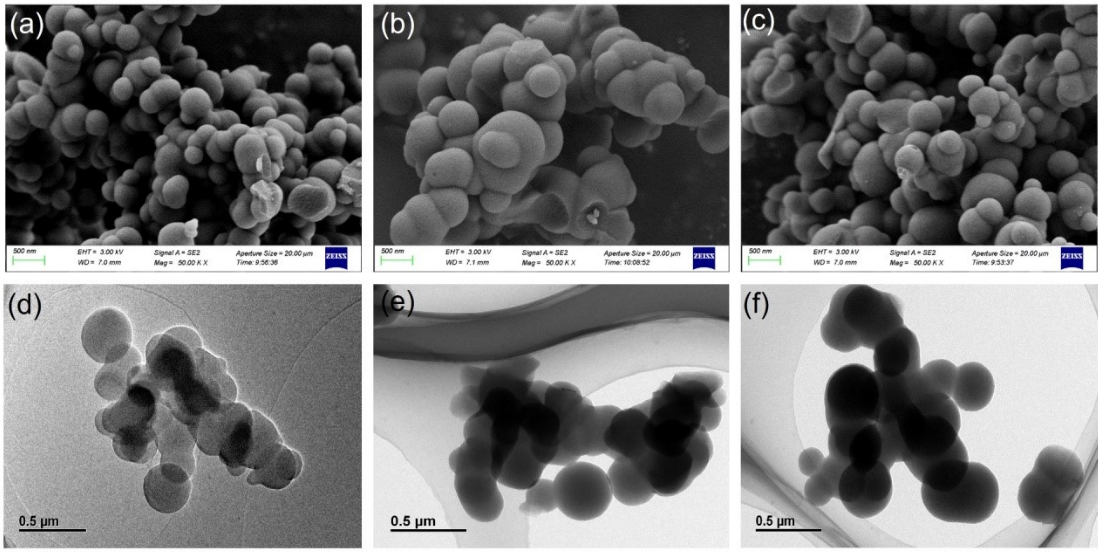

Figure S11. SEM and TEM images of (a, d) TPA-BPy-1, (b, e) TPA-BPy-2, and (c, f) TPA-BPy-3.

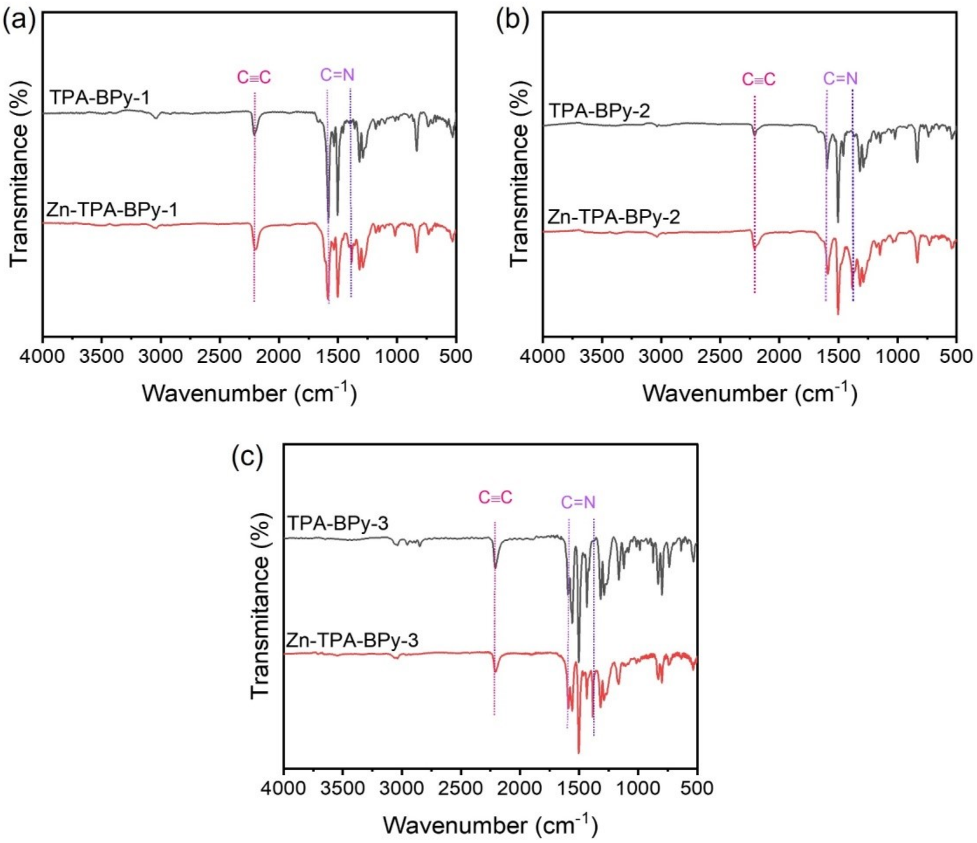

Figure S12. FT-IR spectra of (a) Zn-TPA-BPy-1, (b) Zn-TPA-BPy-2, and (c) Zn-TPA-BPy-3.

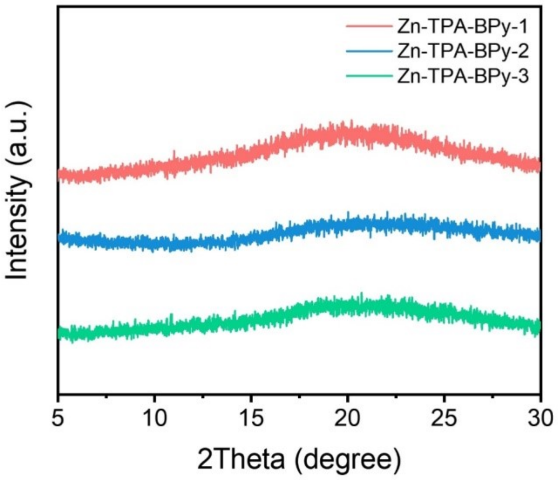

Figure S13. PXRD patterns of Zn-TPA-BPy-1, Zn-TPA-BPy-2, and Zn-TPA-BPy-3.

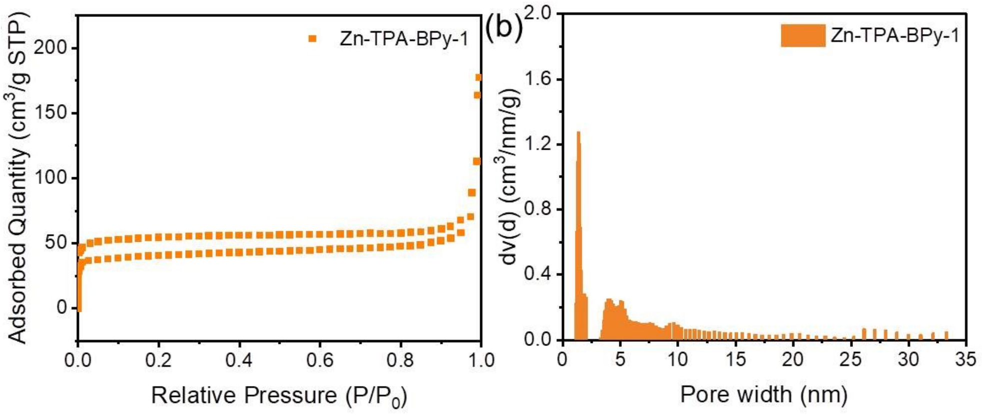

Figure S14. N_2 sorption isotherms of Zn-TPA-BPy-1 and its pore size distribution.

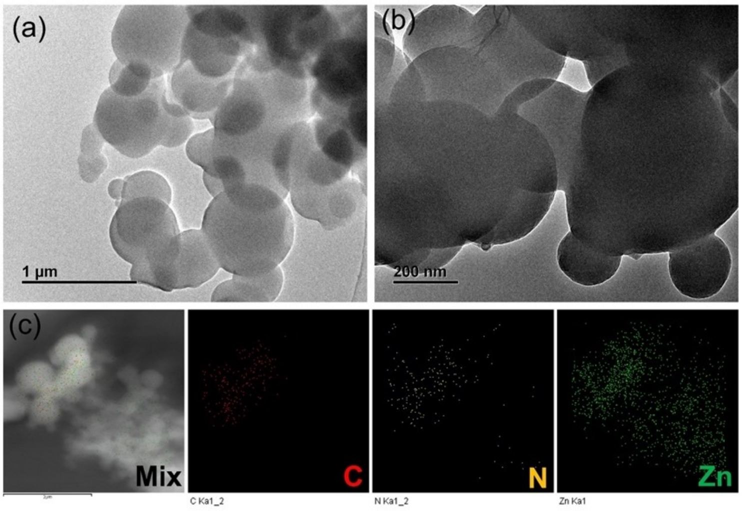

Figure S15. (a, b) TEM images and (c) EDS elemental mapping of Zn-TPA-BPy-1.

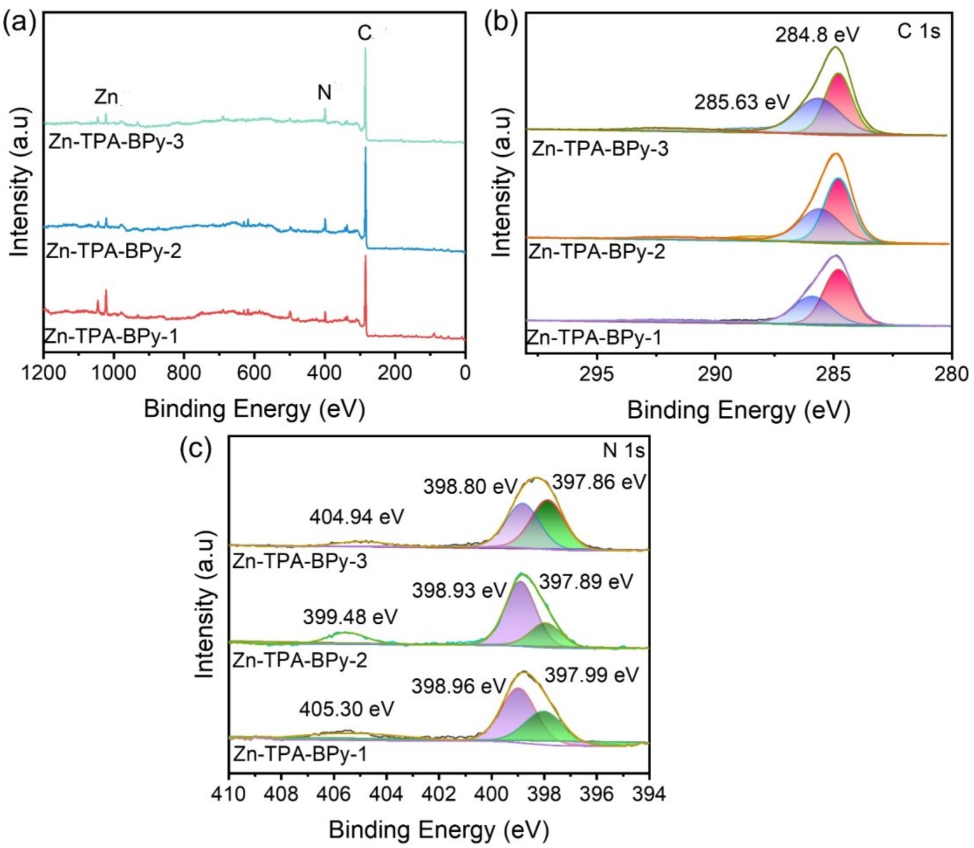

Figure S16. XPS spectra of (a) full survey, (b) C 1s , and (c) N 1s of Zn-TPA-BPy-1, Zn-TPA-BPy-2, and Zn-TPA-BPy-3.

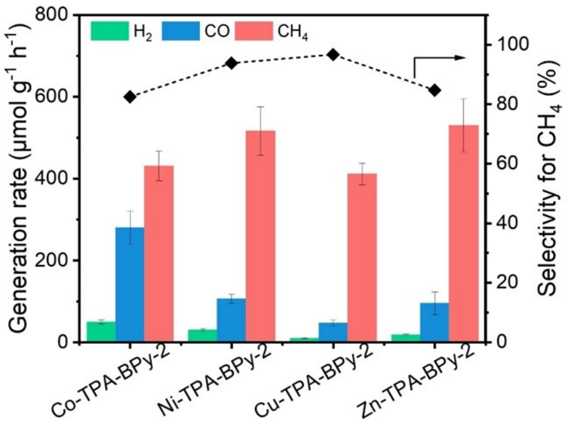

Figure S17. Different metal centers (Co, Ni, Cu, and Zn) in TPA-BPy-2 for photocatalytic CO_2 reduction.

Table S2. Structural parameters of Zn sample as well as Zn foil extracted from the EXAFS fitting. (S_0^2=0.90)

	Shell	C.N.	R (Å)	σ	E_0 (eV)	R-factor (%)
Zn foil	Zn-Zn	6.0	2.64±0.01	0.012±0.001	-0.7±1.3	0.7
ZnO	Zn-O	3.9±0.1	1.96±0.02	0.005±0.001	1.3±1.8	1.3
Zn sample	Zn-N	1.8±0.2	1.98±0.02	0.003±0.003	-0.3±1.9	0.8
	Zn-O	2.2±0.2	2.09±0.02	0.007±0.004		

C.N., coordination number; R, the distance between absorber and backscatter atoms. σ^2 is Debye-Waller factor (a measure of thermal and static disorder in absorber-scatterer distances); ΔE_0 is an edge-energy shift (the difference between the zero-kinetic energy value of the sample and that of the theoretical model).

Table S3. Comparative photocatalysts of CO_2-to-CH_4 conversion from recent reports.

Catalyst	Photosensitizer	Sacrificial agent	CH_4 Products Rate ($\mu mol\cdot g^{-1}\cdot h^{-1}$)	Ref.
CoCo-1	$Ru(phen)_3(PF_6)_2$	TEOA	865	[6]

NST	none	H_2O	147.2	[7]
RT/Cu_2O	none	Gaseous water	0.8	[8]
MOF-808-CuNi	[Ru(bpy)$_3$]Cl_2•6H_2O	TEOA	158.7	[9]
MOF-525-Co	none	TEOA	36.7	[10]
TPA-PQ	none	BNAH/TEA	2150	[11]
Zn-POM	Tir3	0.1 M $KHCO_3$	0.7	[12]
Ag-TiO_2 NPs	none	H_2O	100	[13]
Pd-HN-TiO_2	none	H_2O	237.4	[14]
Au/TiO_2/$W_{18}O_{49}$	none	H_2O	35.55	[15]
Au_1/ZIS	[Ru(bpy)$_3$]Cl_2·6H_2O	TEOA	275	[16]
Cu_3SnS_4	none	H_2O	22.7	[17]
Ov-BC 2	none	H_2O	28.5	[18]
ZnTPP/GO	none	TEOA	41.6	[19]
TPE-PT	none	H_2O	10.6	[20]
In_2S_3/In_2O_3	none	H_2O	16.52	[21]
Zn-TPA-BPy-1	[Ru(bpy)$_3$]Cl_2·6H_2O	TEOA	753.18	**This work**

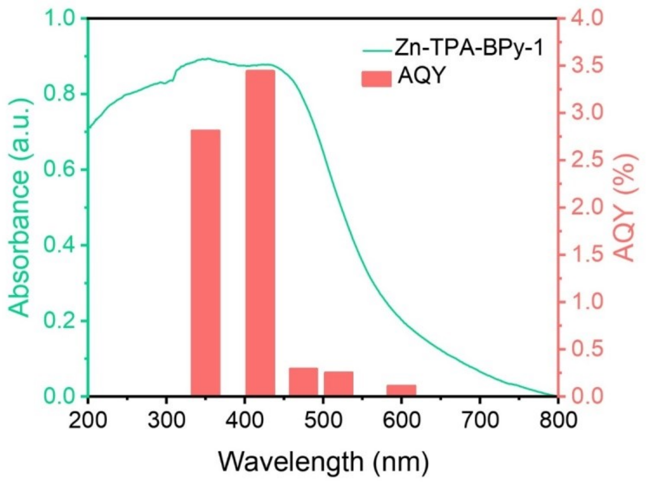

Figure S18. AQY of Zn-TPA-BPy-1 under different monochromatic wavelengths.

Table S4. ICP-OES analysis of Zn-TPA-BPy-n samples.

Sample	Zn content (wt%)	Pd content (wt%)	Cu content (wt%)
Zn-TPA-BPy-1	1.49	0.41	0.12
Zn-TPA-BPy-2	1.57	0.47	0.13
Zn-TPA-BPy-3	1.55	0.46	0.16
Zn-TPA-BPy-3-M	1.79	2.30	0.75

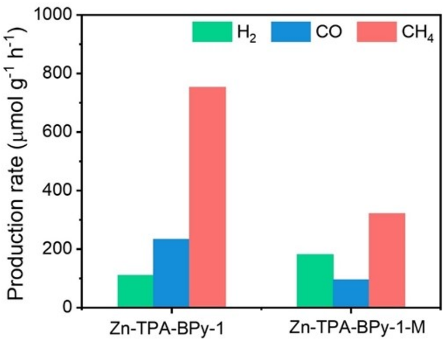

Figure S19. Product generation rates in photocatalytic CO_2 reduction tests with Zn-TPA-BPy-1 and Zn-TPA-BPy-1-M.

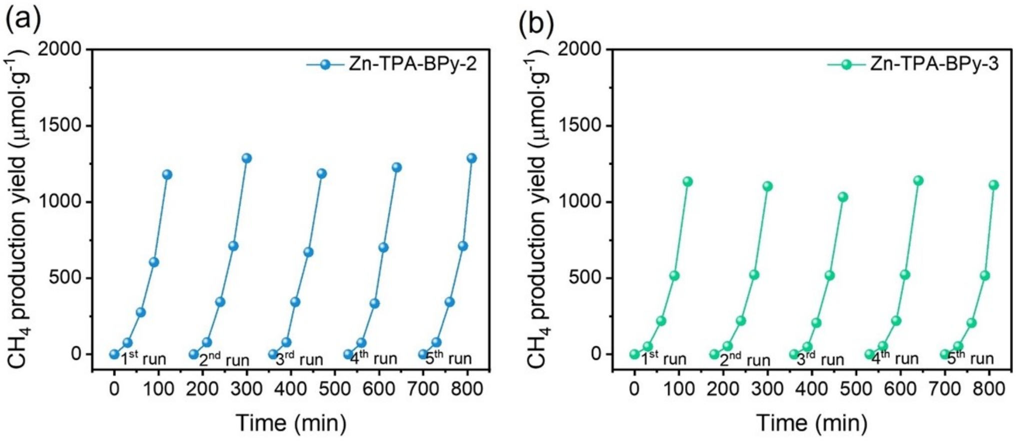

Figure S20. Cycling experiments of (a) Zn-TPA-BPy-2 and (b) Zn-TPA-BPy-3.

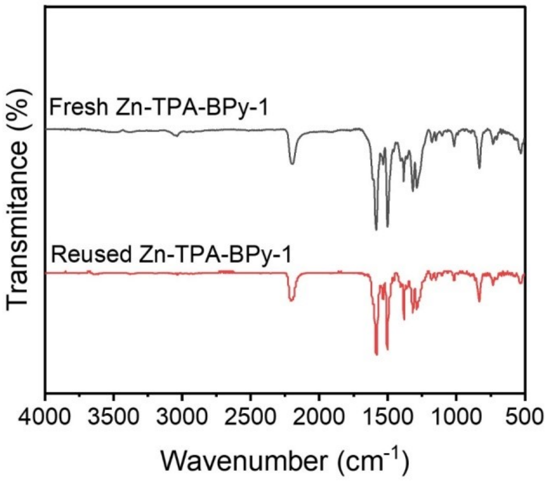

Figure S21. FT-IR spectra of fresh and reused Zn-TPA-BPy-1 catalyst.

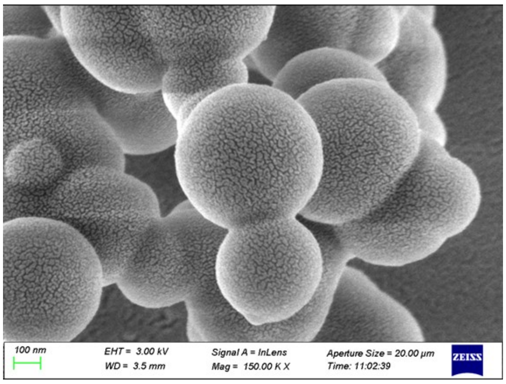

Figure S22. SEM image of reused Zn-TPA-BPy-1 catalyst.

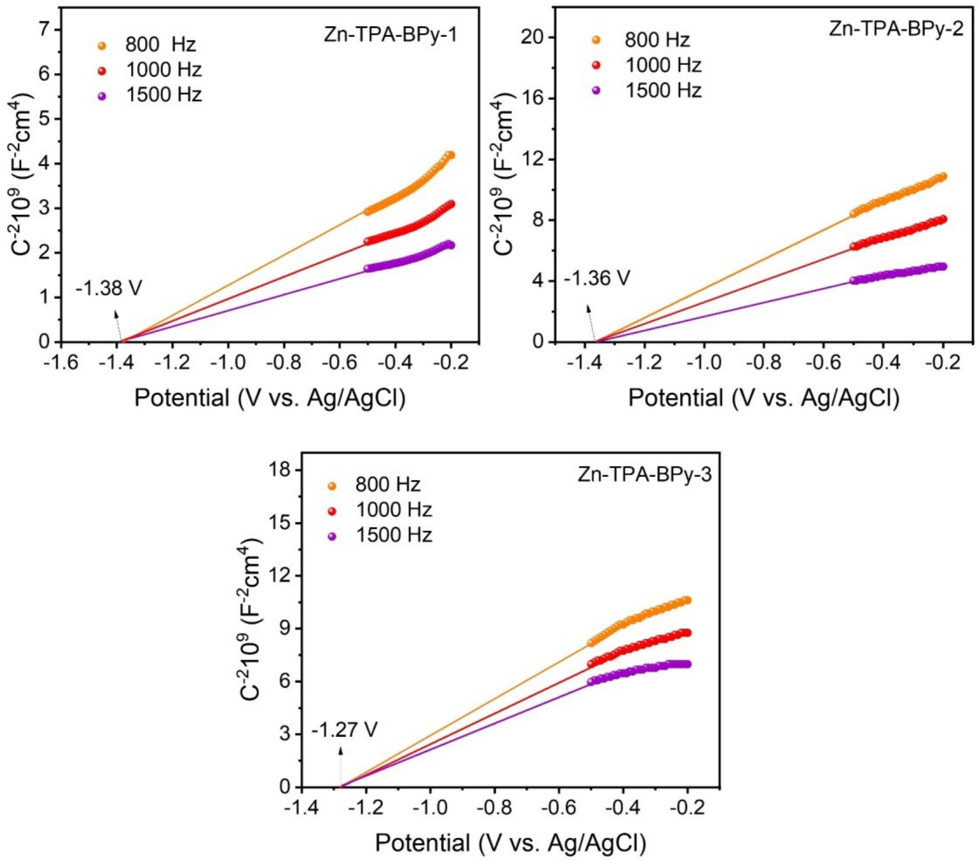

Figure S23. Mott-Schottky plots of as-prepared (a) Zn-TPA-BPy-1, (b) Zn-TPA-BPy-2, and (c) Zn-TPA-BPy-3 samples.

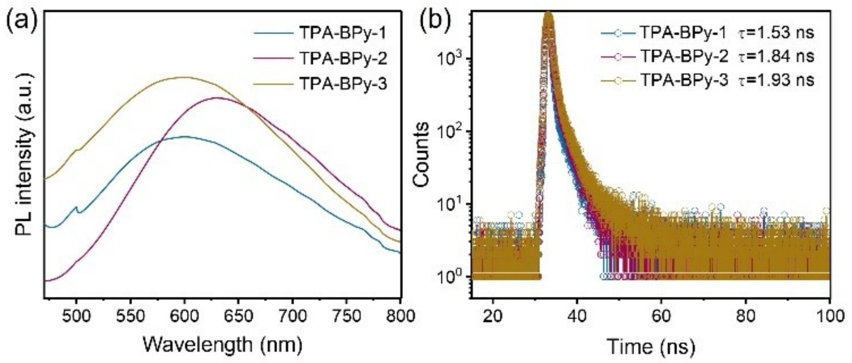

Figure S24. (a) PL spectra and (b) TRPL spectra of TPA-BPy-1, TPA-BPy-2, and TPA-BPy-3 samples.

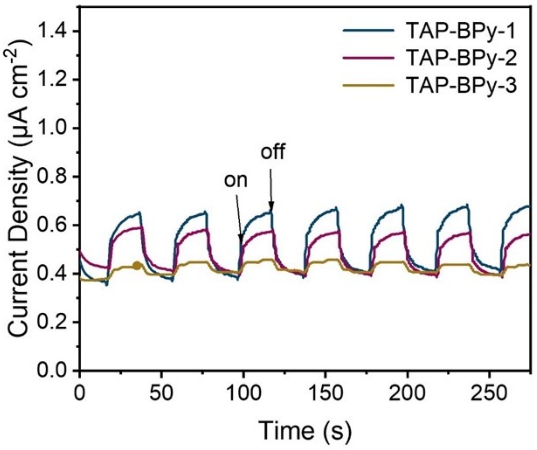

Figure S25. Transient photocurrent responses of the as-prepared TPA-BPy-1, TPA-BPy-2, and TPA-BPy-3 samples.

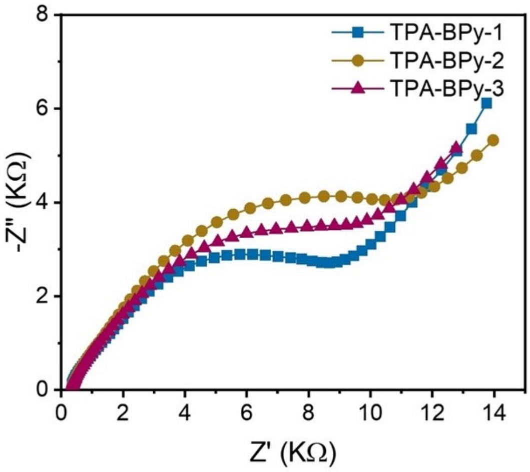

Figure S26. EIS curves of the as-prepared TPA-BPy-1, TPA-BPy-2, and TPA-BPy-3 samples.

Table S5. *D, H,* and S_r indices for measuring electron–hole separation

	D (Å)[a]	*H (Å)*[b]	*Sr*[c]
**S1(Zn-TPA-BPy-1)**	3.336	2.673	0.269
**S1(Zn-TPA-BPy-2)**	2.979	2.881	0.224
**S1(Zn-TPA-BPy-3)**	2.793	2.504	0.263

[a]The distance of charge transfer. [b] Average extension of holes and electrons. [c] Overlap between electron and hole distribution.

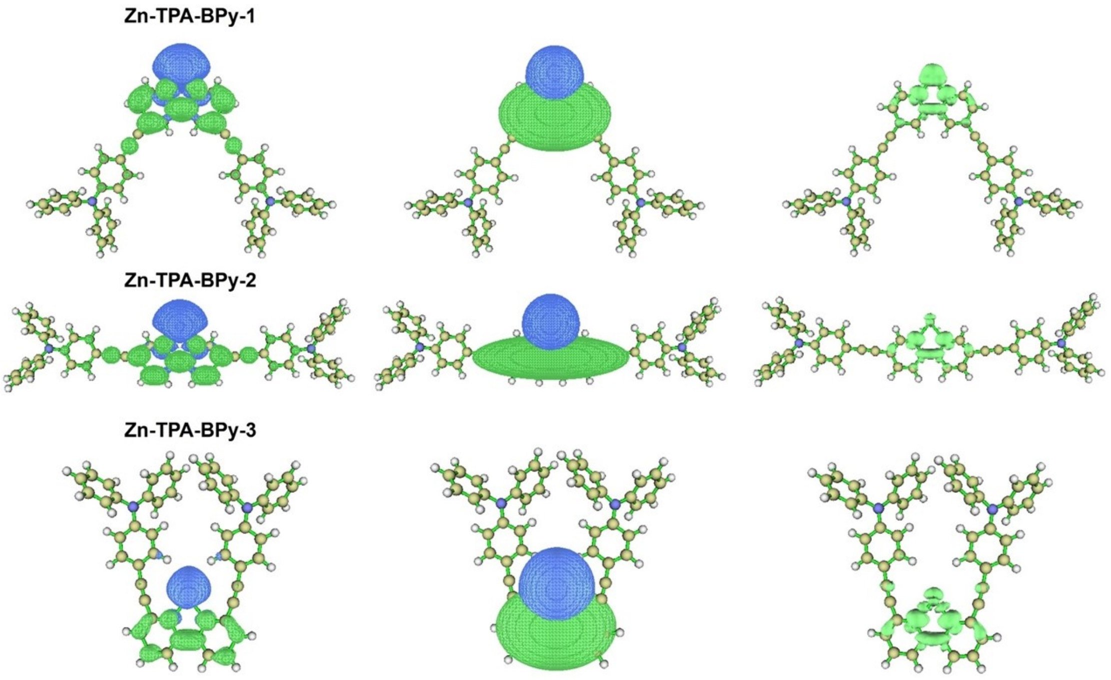

Figure S27. The hole-electron density distributions of S_1 excited states (isovalue = 0.0005 a.u.). The hole (blue) and electron (green) distribution in the excited states (First column). The smoothing description of hole and electron distribution (Second column). The overlap between electron and hole distribution of polymer fragments (Third column).

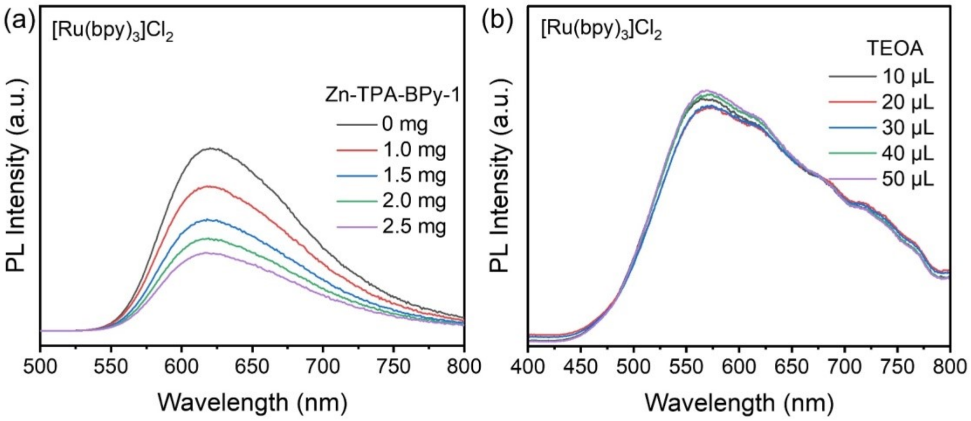

Figure S28. The fluorescence spectra of **Ru** photosensitizer with the different amounts of (a) Zn-TPA-BPy-1 and (b) TEOA.

Reference

[1] Adamo C.; Barone. C. Toward reliable density functional methods without adjustable parameters: The PBE0 model. *J. Chem. Phys.* **1999**, *110*, 6158-6169.

[2] Weigend, F.; Ahlrichs, R. Balanced basis sets of split valence, triple zeta valence and quadruple zeta valence quality for H to Rn: Design and assessment of accuracy. *Phys. Chem. Chem. Phys.* **2005**, *7*, 3297-3305.

[3] Weigend, F. Accurate Coulomb-fitting basis sets for H to Rn. Phys. *Chem. Chem. Phys.* **2006**, *8*, 1057-1065.

[4] Nørskov, J.; Rossmeisl, J.; Logadottir, A.; Lindqvist, L.; Kitchin, J.; Bligaard, T.; Jonsson, H. Origin of the Overpotential for Oxygen Reduction at a Fuel-Cell Cathode. *J. Phys. Chem. B* **2004**, *108*, 17886-17892.

[5] Z. Liu, T. Lu, Q. Chen, An sp-hybridized all-carboatomic ring, cyclo[18]carbon: Electronic structure, electronic spectrum, and optical nonlinearity, *Carbon* **2020**, *165*, 461-467.

[6] Oliver, D.; Luka, D.; Hiroaki, S.; Thomas, J.; Sangji, M.; Kohei, S.; Adam, J.; Samuel, I. Photocatalytic Aqueous CO_2 Reduction to CO and CH_4 Sensitized by Ullazine Supramolecular Polymers. *J. Am. Chem. Soc*. **2022**, *144*, 3127−3136.

[7] Wang, Z.; Wan, Q.; Shi, Y.; Wang, H.; Kang, Y.; Zhu, S.; Lin, S.; Wu, L. Filling COFs with bimetallic nanoclusters for CO_2-to-alcohols conversion with H_2O oxidation. *Appl. Catal. B: Environ.* **2021**, *288*, 120000.

[8] Ali, S.; Lee, J.; Kim, H.; Hwang, Y.; Razzaq, A.; Jung, J.; Cho, C.; x In, C. Sustained, photocatalytic CO_2 reduction to CH_4 in a continuous flow reactor by earth-abundant materials: Reduced titania-Cu_2O Z-scheme heterostructures. *Appl. Catal. B:*

Environ. **2020**, *279*, 119344.

[9] Li, J.; Huang, H.; Xue, W.; Song, X.; Wu, C.; Nie, L.; Li, Y.; Mei, D.; Zhong, C.; Sun, K.; Liu, C.; Pan, Y.; Jiang, H. Self-adaptive dual-metal-site pairs in metal-organic frameworks for selective CO_2 photoreduction to CH_4. *Nat. Catal.* **2021**, *4*, 719–729.

[10] Zhang, H.; Wei, J.; Dong, J.; Liu, G.; Shi, L.; An, P.; Zhao, G.; Kong, J.; Wang, X.; Meng, X.; Zhang, J.; Ye. J. Efficient Visible-Light-Driven Carbon Dioxide Reduction by a Single-Atom Implanted Metal–Organic Framework. *Angew. Chem.* **2016**, *128*, 14522-14526.

[11] Barman, S.; Singh, A.; Rahimi, F.; Maji, T. Metal-Free Catalysis: A Redox-Active Donor–Acceptor Conjugated Microporous Polymer for Selective Visible-Light-Driven CO_2 Reduction to CH_4. *J. Am. Chem. Soc.* **2021**, *143*, 16284-16292.

[12] Kim, N.; Nam, J.; Jo, J.; Seong, J.; Kim, H.; Kwon, Y.; Lah, M.; Lee, J.; Kwon, T.; Ryu, J. Molecular design of heterogeneous electrocatalysts using tannic acid-derived metal–phenolic networks. *Nanoscale*, **2021**, *13*, 20374.

[13] Huang, H.; Shi, R.; Li, Z.; Zhao, J.; Su, C.; Zhang, T. Triphase Photocatalytic CO_2 Reduction over Silver-Decorated Titanium Oxide at a Gas–Water Boundary. *Angew. Chem. Int. Ed.* **2022**, *61*, e202200802.

[14] Zhan, Z.; Wang, H.; Huang, Q.; Li, S.; Yi, X.; Tang, Q.; Wang, J.; Tan, B. Grafting Hypercrosslinked Polymers on TiO_2 Surface for Anchoring Ultrafine Pd Nanoparticles: Dramatically Enhanced Efficiency and Selectivity toward Photocatalytic Reduction of CO_2 to CH_4. *Small* **2022**, *18*, 2105083.

[15] Jiang, X.; Huang, J.; Bi, Z.; Ni, W.; Gurzadyan, G.; Zhu, Y.; Zhang, Z. Plasmonic Active "Hot Spots"-Confined Photocatalytic CO_2 Reduction with High Selectivity for CH_4 Production. *Adv. Mater.* **2022**, *34*, 2109330.

[16] Si, S.; Shou, H.; Mao, Y.; Bao, X.; Zhai, G.; Song, K.; Wang, Z.; Wang, P.; Liu, Y.; Zheng, Z.; Dai, Y.; Song, L.; Huang, B.; Cheng, H. Low-Coordination Single Au Atoms on Ultrathin $ZnIn_2S_4$ Nanosheets for Selective Photocatalytic CO_2 Reduction towards CH_4. *Angew. Chem. Int. Ed.* **2022**, *61*, e202209446.

[17] Wang, J.; Bo, T.; Shao, B.; Zhang, Y.; Jia, L.; Tan, X.; Zhou, W.; Yu, T. Effect of S vacancy in Cu_3SnS_4 on high selectivity and activity of photocatalytic CO_2 reduction. *Appl. Catal. B: Environ.* **2021**, *297*, 120498.

[18] Fan, J.; Shi, L.; Ge, H.; Liu, J.; Deng, X.; Li, Z.; Liang, Q. Regulating the Oxygen Vacancy on Bi_2MoO_6/Co_3O_4 Core-Shell Nanocage Enables Highly Selective CO_2 Photoreduction to CH_4. *Adv. Funct. Mater.* **2024**, 2412078.

[19] He, Y.; Wang, Z.; Cao, A.; Xu, X.; Li, J.; Zhang, B.; Kang, L. Construction of graphene oxide-coated zinc tetraphenyporphyrin nanostructures for photocatalytic CO_2 reduction to highly selective CH_4 product. *J. Colloid Interf. Sci.* **2023**, *638*, 123–134.

[20] Zou, W.; Cheng, Y.; Ye, Y.; Wei, X.; Tong, Q.; Dong, L.; Ouyang. G. Metal-Free Photocatalytic CO_2 Reduction to CH_4 and H_2O_2 under Non-sacrificial Ambient Conditions. *Angew. Chem. Int. Ed.* **2023**, *62*, e202313392.

[21] Lai, K.; Sun, Y.; Li, N.; Gao, Y.; Li, H.; Ge, L.; Ma, T. Photocatalytic CO_2-to-CH_4 Conversion with Ultrahigh Selectivity of 95.93% on S-Vacancy Modulated

Spatial In_2S_3/In_2O_3 Heterojunction. *Adv. Funct. Mater.* **2024**, 2409031.
